# Temperature Effect on Rhizome Development in Perennial rice

**DOI:** 10.1186/s12284-024-00710-2

**Published:** 2024-05-08

**Authors:** Kai Wang, Jie Li, Yourong Fan, Jiangyi Yang

**Affiliations:** State Key Laboratory for Conservation and Utilization of Subtropical Agro-Bioresources, College of Life Science and Technology, Guangxi University, Nanning, 530004 China

**Keywords:** Bud outgrowth, Gravitropism, Rhizome, Thermo-morphogenesis

## Abstract

Traditional agriculture is becoming increasingly not adapted to global climate change. Compared with annual rice, perennial rice has strong environmental adaptation and needs fewer natural resources and labor inputs. Rhizome, a kind of underground stem for rice to achieve perenniallity, can grow underground horizontally and then bend upward, developing into aerial stems. The temperature has a great influence on plant development. To date, the effect of temperature on rhizome development is still unknown. Fine temperature treatment of *Oryza longistaminata* (*OL*) proved that compared with higher temperatures (28–30 ℃), lower temperature (17–19 ℃) could promote the sprouting of axillary buds and enhance negative gravitropism of branches, resulting in shorter rhizomes. The upward growth of branches was earlier at low temperature than that at high temperature, leading to a high frequency of shorter rhizomes and smaller branch angles. Comparative transcriptome showed that plant hormones played an essential role in the response of *OL* to temperature. The expressions of *ARF17*, *ARF25* and *FucT* were up-regulated at low temperature, resulting in prospectively asymmetric auxin distribution, which subsequently induced asymmetric expression of *IAA20* and *WOX11* between the upper and lower side of the rhizome, further leading to upward growth of the rhizome. Cytokinin and auxin are phytohormones that can promote and inhibit bud outgrowth, respectively. The auxin biosynthesis gene *YUCCA1* and cytokinin oxidase/dehydrogenase gene *CKX4* and *CKX9* were up-regulated, while cytokinin biosynthesis gene *IPT4* was down-regulated at high temperature. Moreover, the *D3* and *D14* in strigolactones pathways, negatively regulating bud outgrowth, were up-regulated at high temperature. These results indicated that cytokinin, auxins, and strigolactones jointly control bud outgrowth at different temperatures. Our research revealed that the outgrowth of axillary bud and the upward growth of *OL* rhizome were earlier at lower temperature, providing clues for understanding the rhizome growth habit under different temperatures, which would be helpful for cultivating perennial rice.

**Supplementary Information:**

The online version contains supplementary material available at 10.1186/s12284-024-00710-2.

## Background

Plant growth is affected by multiple environmental factors, such as light, temperature, moisture, carbon dioxide and soil conditions (Liu et al. 2022a). Global warming and frequent occurrence of extreme weather have brought negative effects on the sustainable development of agriculture (Anderson et al. 2016; Coumou and Rahmstorf 2012; Fuglie 2021). Annual crops are widely planted in modern agriculture, but existing annual cultivars are difficult to adapt to continual climate change (Altieri et al. 2015; Chapman et al. 2022). Tillage is necessary before cultivating annual crops every year, and frequent tillage increases the risk of soil erosion (Glover et al. 2010). Without the cover of vegetation, carbon elements are easily emitted into the atmosphere from the soil, which exacerbates the greenhouse effect (Chapman et al. 2022). In natural ecosystems, perennials with better environmental adaptability tend to be more dominant than annuals (Chapman et al. 2022; Li et al. 2022b). Compared with annual crops, perennials can live for several years and seasons, avoiding repeated tillage and requiring fewer resources and labor inputs (Larson et al. 2014; Zhang et al. 2023). Perennials have deeper and well-developed root system, decreasing the risk of soil erosion and reducing CO_2_ emission into the atmosphere, which can help to retain more carbon in the soil over time (Crews et al. 2018). *O. longistaminata* (*OL*) is a kind of perennial wild rice with vigorous rhizomes, widely distributed in Africa (Hu et al. 2003). Among several rhizomatous wild rice, only *OL* has the same “AA” type genome as cultivated rice (Hu et al. 2003; Tao and Sripichitt 2000) and is well resistant to biotic and abiotic stresses, making it an ideal genetic resource for the improvement of cultivated rice (He et al. 2014; Hu et al. 2003; Sacks et al. 2003; Tao and Sripichitt 2000). The rhizome is a kind of underground stem for vegetative propagation, which can enable plants to live for years (Guo et al. 2021; Li et al. 2022b; Shibasaki et al. 2021). The rhizome can store energy and nutrients that support plants to survive through unfriendly environments such as cold and drought (He et al. 2014; Paterson et al. 1995; Westerbergh and Doebley 2004). Recent studies have shown that the development of rice rhizome is controlled by multiple genes (Fan et al. 2020; Li et al. 2022a).

The gravity is one of the most important environment factors, and plant organs can adjust their growth directions in response to gravity (Morita and Tasaka 2004; Strohm et al. 2012). The plant organs can maintain their growth orientation in response to gravity and redirect their growth orientation through gravitropic response when the growth orientation deviates from the appropriate direction (Nakamura et al. 2019). The downward growth of roots shows positive gravitropism, and the upward growth of shoots is opposite to the direction of gravity, showing negative gravitropism (Morita and Tasaka 2004; Nakamura et al. 2019). Rhizome can grow horizontally under the ground, and then exhibit negative gravitropism, bending upward and growing into new plants (Gizmawy et al. 1985; Guo et al. 2021). The upward growth of rhizome is related to the asymmetric growth between the lower and upper sides of it. When the upward growth of rhizome starts, cells grow faster in the lower side of rhizome bent area than those in the upper side (Bessho-Uehara et al. 2018). Gravitropism can be divided into sequential steps: gravity sensing, signal production in the gravity-perception cell, signal transmission, asymmetric auxin distribution and asymmetric cell growth between the upper and the lower side of the gravity-responding organs (Morita and Tasaka 2004; Strohm et al. 2012; Tasaka et al. 1999). Recently, the research on rice has shown that the shoot branch angle is closely related to shoot gravitropism (Hu et al. 2020; Huang et al. 2021; Li et al. 2020b, 2021). The defect of plant responding to gravity will cause a large branching angle (Huang et al. 2021; Yoshihara and Spalding 2017; Zhang et al. 2018). The shoot gravitropism of rice is related to the asymmetric distribution of auxin, and the *IAA20*, *WOX6* and *WOX11* genes are expressed asymmetrically in response to auxin, allowing rice to adjust growth direction in response to gravitropism (Hu et al. 2020; Li et al. 2020b; Zhang et al. 2018).

Rhizome of *OL* is initially developed from the axillary bud at the shoot base of the seedlings (Fan et al. 2017), and the rhizome development of *OL* is related to the outgrowth of bud (Shibasaki et al. 2021). The process of axillary buds developing into new branches consists of axillary buds being activated from dormancy and continuous growth of buds, which is controlled by the interaction of multiple environmental factors and endogenous ones including hormones (Ongaro and Leyser 2007; Rameau et al. 2015). The shoot branching in plants is mainly controlled by auxin, cytokinin and strigolactones, all of which playing major roles in outgrowth of bud (Kotov et al. 2021; Rameau et al. 2015). Auxin is first identified to be related to apical dominance, inhibiting the outgrowth of lateral bud in intact plants (Ongaro and Leyser 2007; Rameau et al. 2015). Instead, cytokinin can break the dormancy of buds and promote their growth (Sachs and Thimann 1967; Wickson and Thimann 1958). Strigolactones, a newly discovered plant hormone, can also inhibit shoot branching (Gomez-Roldan et al. 2008; Umehara et al. 2008). Strigolactones can promote the expression of *CKX9*, a *CYTOKININ OXIDASE/DEHYDROGENASE* (*CKX*) that directly destroys cytokinin activity (Duan et al. 2019). Cytokinin acts downstream of auxin (Rameau et al. 2015). Auxin can reduce the content of cytokinin by inhibiting the expression of cytokinin synthesis gene *IPT* (Tanaka et al. 2006; Zhang et al. 2010) and promoting the expression of *CKXs* to destroy the activity of cytokinin (Carabelli et al. 2007; Gao et al. 2014).

Temperature is one of the major environmental factors affecting the growth and development of the plants (Gong et al. 2020). Temperature can affect the shoot gravitropism in plants: both low and high temperatures can attenuate gravitropism of inflorescence stems in *Arabidopsis* (Fukaki et al. 1996; Kim et al. 2016). Compared with spring types, there is a higher frequency of prostrate growth (larger branch angle) in winter durum wheat genotypes, and the frequency of prostrate growth is higher at lower temperature than that at higher temperature, suggesting that temperature can affect the branch angle of winter durum (Marone et al. 2020). Environmental temperature can also affect the outgrowth of axillary bud (Sánchez et al. 2014). During the early stage of rice after sowing (three-five weeks after sowing), four temperature groups including 22 ℃, 25 ℃, 28 ℃ and 31 ℃ were used to identify how temperature affected tillering, and tiller number was found to increase mostly at 28 ℃ (Yoshida 1973).

Rhizome is the key organ that can help to breed perennial crops. Until now, the effect of temperature on *OL* rhizome development is still unknown. We found that the gravitropic responses of rhizome and the sprouting of axillary bud were greatly affected by temperature, and plant hormones played an important role in these responses. Our findings will be helpful for understanding the development of rhizome at different temperatures.

## Results

### The Development of Aerial stem from the Rhizome

**Fig. 1:**
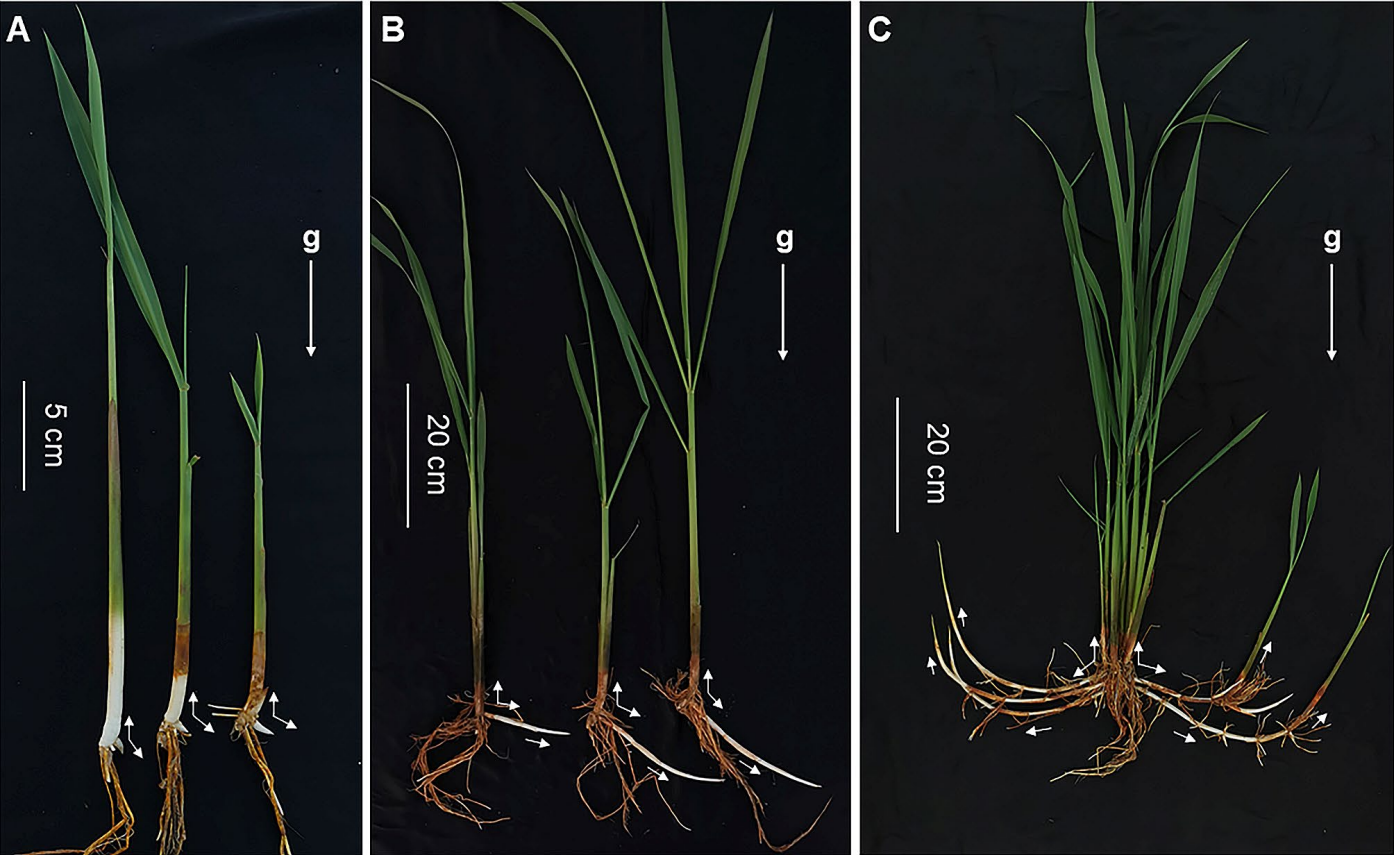
The progress that axillary buds of *OL* develop into rhizome and aerial stem. **A** The mother plants with sprouted axillary bud. **B** The horizontal growth of rhizomes before the start of upward growth. **C** The rhizomes bend upward and develop into aerial stems. The white arrow represents the growth direction of the rhizome relative to the mother plant, and the arrow marked with “g” indicates the direction of gravity

The rhizome of *OL*, a root-like lateral branch, is developed from the axillary bud of the mother plant (Fig. 1). Several initially sprouted basal axillary buds of *OL* (Fig. 1A) could keep downward and horizontal growth for a distance before upward growth (Fig. 1B), and the rhizome would eventually bend up and then grow out of the soil to become a daughter plant (also named ramet) (Fig. 1C). During this process, the growth direction of the rhizome gradually changed over time. The upward growth of the rhizome was opposite to the direction of the gravity, exhibiting negative gravitropism. But before upward growth, the rhizome exhibited diagravitropism, somewhat like the stolon. The difference in growth direction resulted in a certain angle between the branches and their mother plants (Fig. 1). In a word, the response of the rhizome to the gravity was related to the process that rhizome developed into the aerial stem.

### The Effect of high/low Temperature on the Sprouting of Axillary Bud

**Fig. 2:**
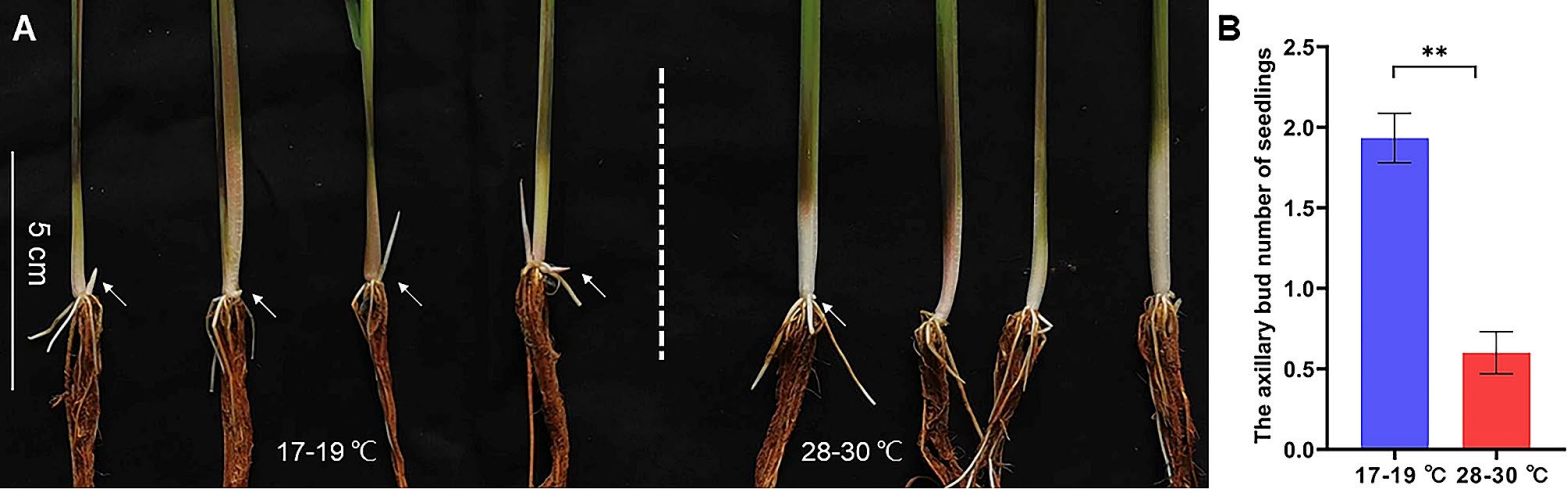
The axillary bud outgrowth of *OL* seedlings at 17–19 ℃ and 28–30 ℃. **A** The seedlings cultured in paddy soil for 12 days. **B** The average number of sprouted axillary buds per seedling at 17–19 ℃ and 28–30 ℃ for 12 days. Values are mean ± se (*n* = 15). The statistical significance is determined by Student’s *t*-test; **, *P* < 0.01. The white arrows represent new branches (tillers or rhizomes)

Seedlings of *OL* about 3–4 leaf stage (axillary buds not sprouted) were planted in growth chambers with the high (28–30 ℃) and low (17–19 ℃) temperature, respectively. Our main concern was the effect of temperature on the development of axillary buds. Several days later, these axillary buds will develop into tillers or rhizomes. Seedlings were cultured in the rice paddy soil at 17–19 ℃, and the axillary buds of seedlings began to sprout after 5–7 days. Compared with the effect of low temperature, bud sprouting was delayed by 3–7 days at 28–30 ℃ (Fig. 2A), and the average number of sprouted axillary buds owned by per seedling was less than that at low temperature (Fig. 2B). In order to dynamically observe the sprouting of axillary buds, we repeated temperature treatment by hydroponics. Similar to that in soil culture, axillary buds cultured in hydroponics at 17–19 ℃ also began to sprout within 5–7 days, while delayed by 2–5 days at 28–30 ℃ (Additional file 1: Fig. S1A). The average number of sprouted axillary bud at high temperature was also less than that at low temperature (Additional file 1: Fig. S1B). These results showed that the sprouting of axillary buds was earlier at 17–19 ℃ than that at 28–30 ℃. It is inferred that lower temperatures could contribute to break the dormancy of the axillary buds, leading to their earlier outgrowth. Besides the effect of temperature on sprouting of the axillary buds, we found that the seedlings were taller at 28–30 ℃ than that at 17–19 ℃, cultured either in the paddy soil or hydroponics (Additional file 1: Fig. S2).

### The Effect of Temperatures on Rhizome Length and Shoot Branch Angle

Unlike the tiller, the main characteristic of the rhizome is that they can initially grow horizontally underground and then change their growth direction, bending upward and developing into aerial stem (Bessho-Uehara et al. 2018; Guo et al. 2021). During the development process of rhizome, the final growth direction of the rhizome was significantly different from the initial direction (Fig. 1). We found that the earlier of the rhizome developing into aerial stems, the shorter of the rhizome. On the other side, the later of the rhizome developing into aerial stems, the longer of the rhizome (Fig. 1C). So, the branch angle between the mother plant and the tiller/rhizome and the rhizome length were used to evaluate the effect of the temperature on rhizome development in our following research.

To identify the effect of different temperatures on rhizome development, hydroponic seedlings were treated with four temperatures including 20–22 ℃, 25–27 ℃, 28–30 ℃ and 30–32 ℃ (Additional file 1: Fig. S3A-D), referring to the temperature range for rice growth (Sánchez et al. 2014). The rhizome length and the branch angle were measured after about three-four weeks. The branch angle was smaller, and the rhizome was shorter at 20–22 ℃ than those at higher temperatures, and there was a growing trend for branch angle and branch length (except for branch length at 30–32 ℃ due to delayed bud sprouting at this temperature) with temperature rising (Additional file 1: Fig. S3E-F). The sprouting of axillary bud was excessively delayed at 30–32 ℃, resulting in shorter rhizome length than those at other temperature groups (Additional file 1: Fig. S3F).

Carbohydrates play an important role in the growth of rhizomes and increasing sucrose concentration will delay the upward growth of rhizomes (Bessho-Uehara et al. 2018; Fan et al. 2017; Fan et al. 2022). It has been reported that 80 g/L concentration of sucrose in solid medium was well suitable for rhizome growth (Fan et al. 2022). The rate of photosynthesis in plants is affected by temperature, and lower temperature will reduce the efficiency of photosynthesis (Moore et al. 2021), so the different effect of the high and low temperatures on rhizome (bud) angle maybe due to their effect on photosynthate (sucrose) production. To reduce the effect of photosynthate (sucrose) on rhizome (bud) angle, seedlings were cultured in solid medium with 80 g/L sucrose to explore the relationship between temperature and branch angle. The low and high temperature was set 20–22℃ and 28–30℃, separately (Additional file 1: Fig. S4A). The branch angle was still smaller at 20–22 ℃ than that at 28–30 ℃ (Additional file 1: Fig. S4B), which implied that temperature could directly affect the branch angle, although the rates of photosynthesis were different at low and high temperature.

**Fig. 3:**
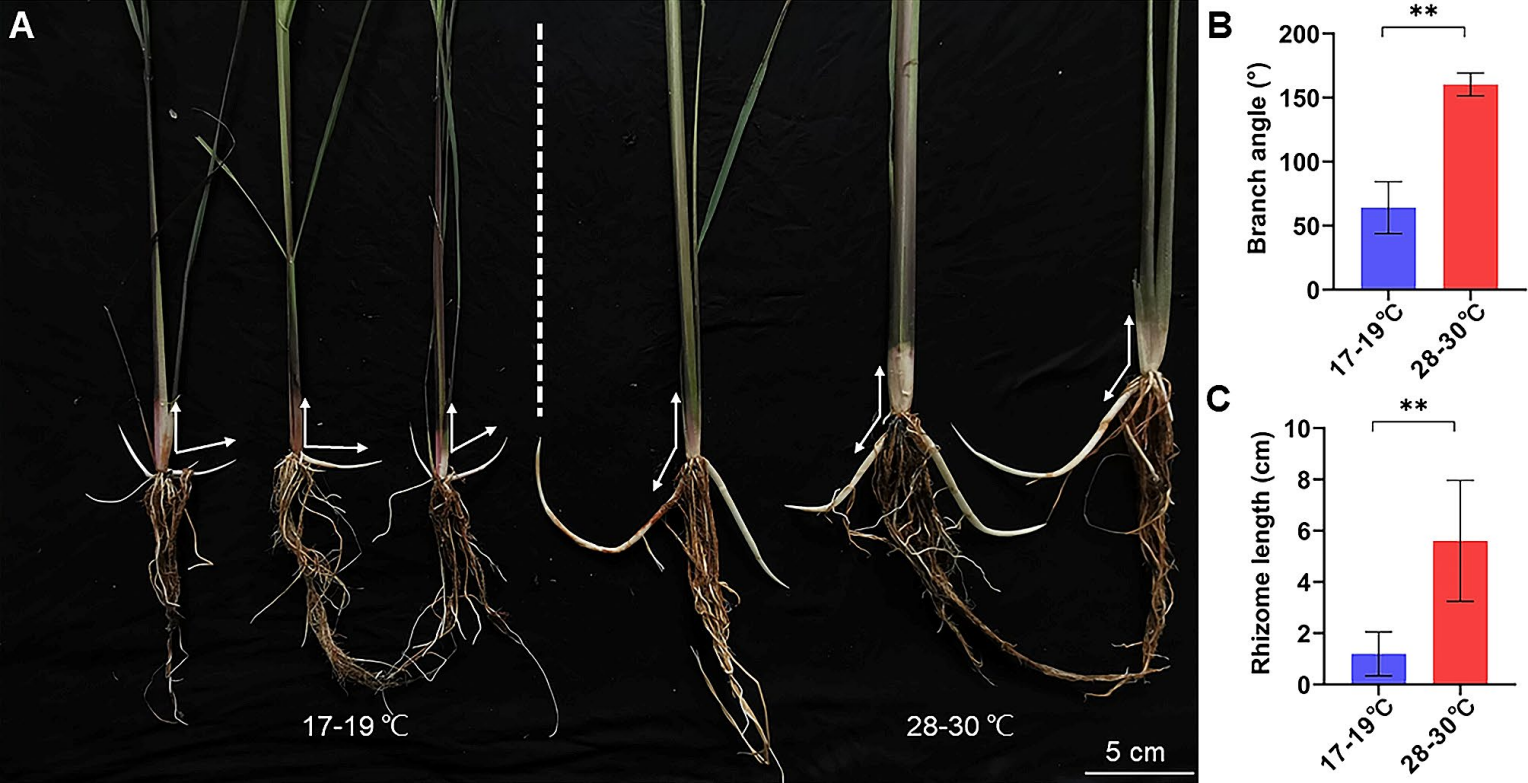
The rhizome growth of *OL* seedlings cultured at 17–19 ℃ and 28–30 ℃. **A** The branch of seedlings cultured at 17–19 ℃ and 28–30 ℃. **B** The average branch angle at 17–19 ℃ and 28–30 ℃. **C** The average rhizome length at 17–19 ℃ and 28–30 ℃. Values are mean ± sd (*n* = 20). The statistical significance is determined by Student’s *t*-test; **, *P* < 0.01. The white arrow represents the angle between the branch and mother plant

To simulate the natural environment, seedlings were planted in paddy soil, and 20–22 ℃ and 28–30 ℃ were initially set as the low and high temperatures in growth chambers (Additional file 1: Fig. S5A). Unlike the results of seedlings cultured in hydroponic, there was no significant difference in branch angle between 20 and 22 ℃ and 28–30 ℃ in paddy soil (Additional file 1: Fig. S5B), which may be due to the physical and chemical properties of the soil. So, the lower temperature 17–19 ℃ was selected for further soil culture. Immediately after the first axillary bud began to sprout, the seedlings were selected for soil culture at 17–19 ℃ and 28–30 ℃ separately, and the branch angle was measured after about a week. Compared with 28–30 ℃, the branch angle was smaller at 17–19 ℃ (Additional file 1: Fig. S6A). The average bud angle was about 62° at 17–19 ℃, while about 148° at 28–30 ℃ (Additional file 1: Fig. S6B). These results indicated that the branch angle was greatly influenced by environmental temperature. These seedlings were further cultured for about three to four weeks until the axillary buds had developed into rhizomes or tillers (Fig. 3A). The average branch angle reached about 64° at 17–19 ℃ and about 160° at 28–30 ℃, respectively (Fig. 3B). The average rhizome length reached about 1.2 cm at 17–19 ℃ and about 5.6 cm at 28–30 ℃ (Fig. 3C). At the same culturing time interval, the branches were shorter at low temperature than that at high temperature (Fig. 3). These results suggested that the growth of the axillary buds was faster at high temperature than that at low temperature (Fig. 3; Additional file 1: Fig. S6).

Compared with the higher temperature, the low temperature could enhance negative gravitropism of the rhizome, resulting in earlier upward growth of branch. These results suggested that the branch angle was more stable during elongation at high temperature than that at low temperature.

### Transcriptome Sequencing Analysis and Validation

**Fig. 4:**
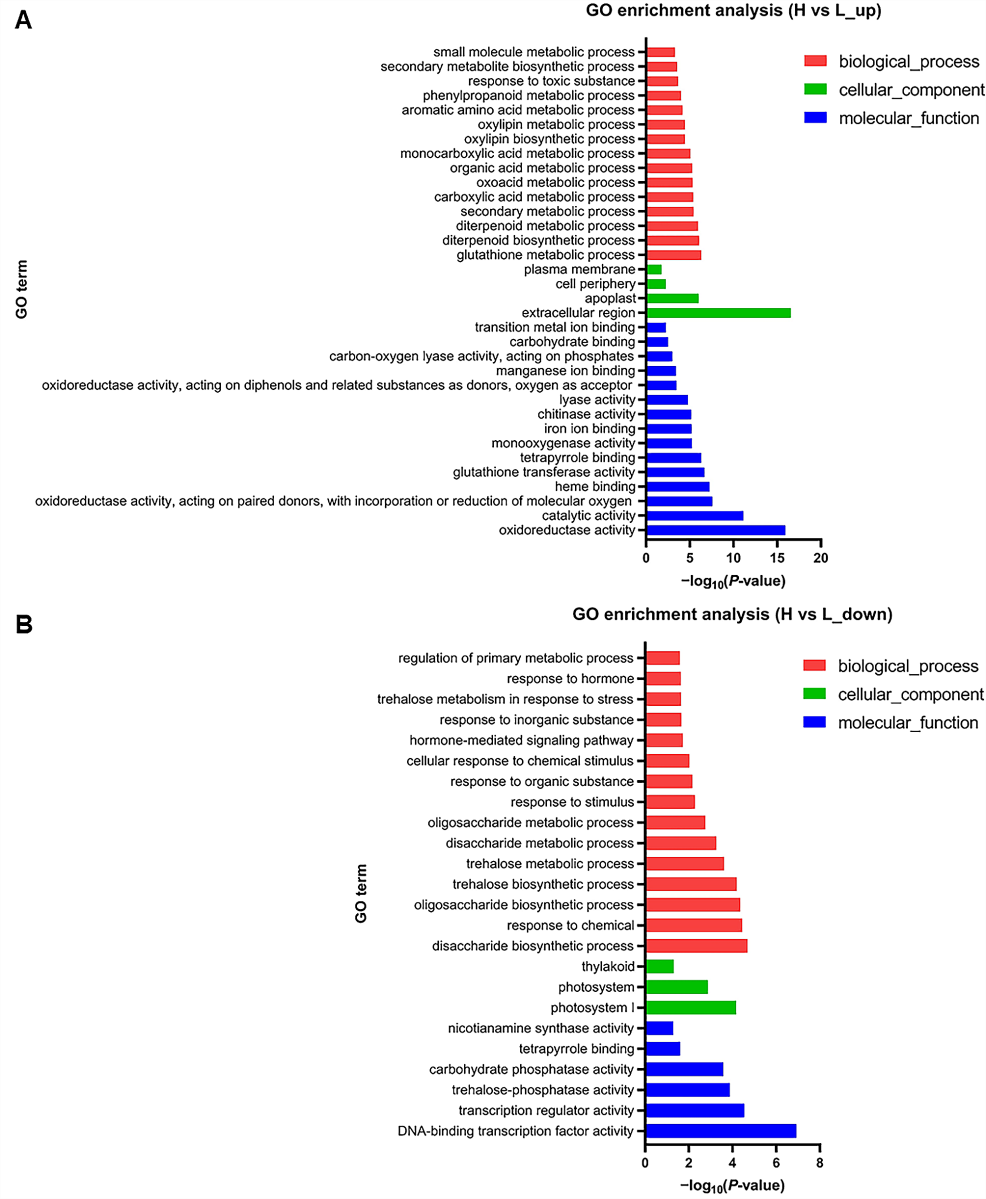
GO enrichment analysis. **A** GO enrichment analysis of genes that up-regulated expression in “H vs. L”. **B** GO enrichment analysis of genes that down-regulated expression in “H vs. L”. The vertical axis indicates GO term; the horizontal axis indicates -log_10_(*P*-value). Top15 enrichment results of biological process, cellular component and molecular function are separately displayed based on the premise of *p-adjust* < 0.05

Based on the temperature response results, the high temperature (28–30 ℃) was more favorable for rhizome growth than the low temperature (17–19 ℃) in *OL*. To reveal the molecular mechanism, the crowns (shortened basal internode with axillary bud) of *OL* seedlings were collected for transcriptome sequencing. These seedlings were cultured in plant chamber for about five days in soil, and the 17–19 ℃ and 28–30 ℃ were set as the low and high temperatures, respectively. The Venn diagram showed that 834 genes and 1240 genes were specifically expressed under the high temperature and the low temperature, respectively, while 20,133 genes were expressed under both temperatures (Additional file 1: Fig. S7A). Differentially expressed genes (DEGs) between the high temperature (28–30 ℃) and the low temperature (17–19 ℃) (abbreviated as H vs. L) showed that 2107 genes were up-regulated (Additional file 1: Fig. S7B; Additional file 2: Table S1) and 1783 genes were down-regulated (Additional file 1: Fig. S7B; Additional file 2: Table S2). The up-regulated and down-regulated genes in “H vs. L”, were separately selected for GO and KEGG enrichment analysis. The genes with up-regulated expression in “H vs. L” were mainly related to the difference of metabolic processes (Fig. 4A). The response to hormone and hormone-mediated signaling pathway were found in the genes with down-regulated expression in “H vs. L” (Fig. 4B), and both pathways were related to adapting to the environment changes. Plants are exposed to different environmental changes all the time and they need to adjust their development to adapt to those changes in time, and plant hormones play an important role in the process (Benková 2016). Previous studies have shown that plant hormones are involved in the regulation of axillary bud outgrowth and shoot gravitropism (Li et al. 2020b; Rameau et al. 2015; Zhang et al. 2018).

**Fig. 5:**
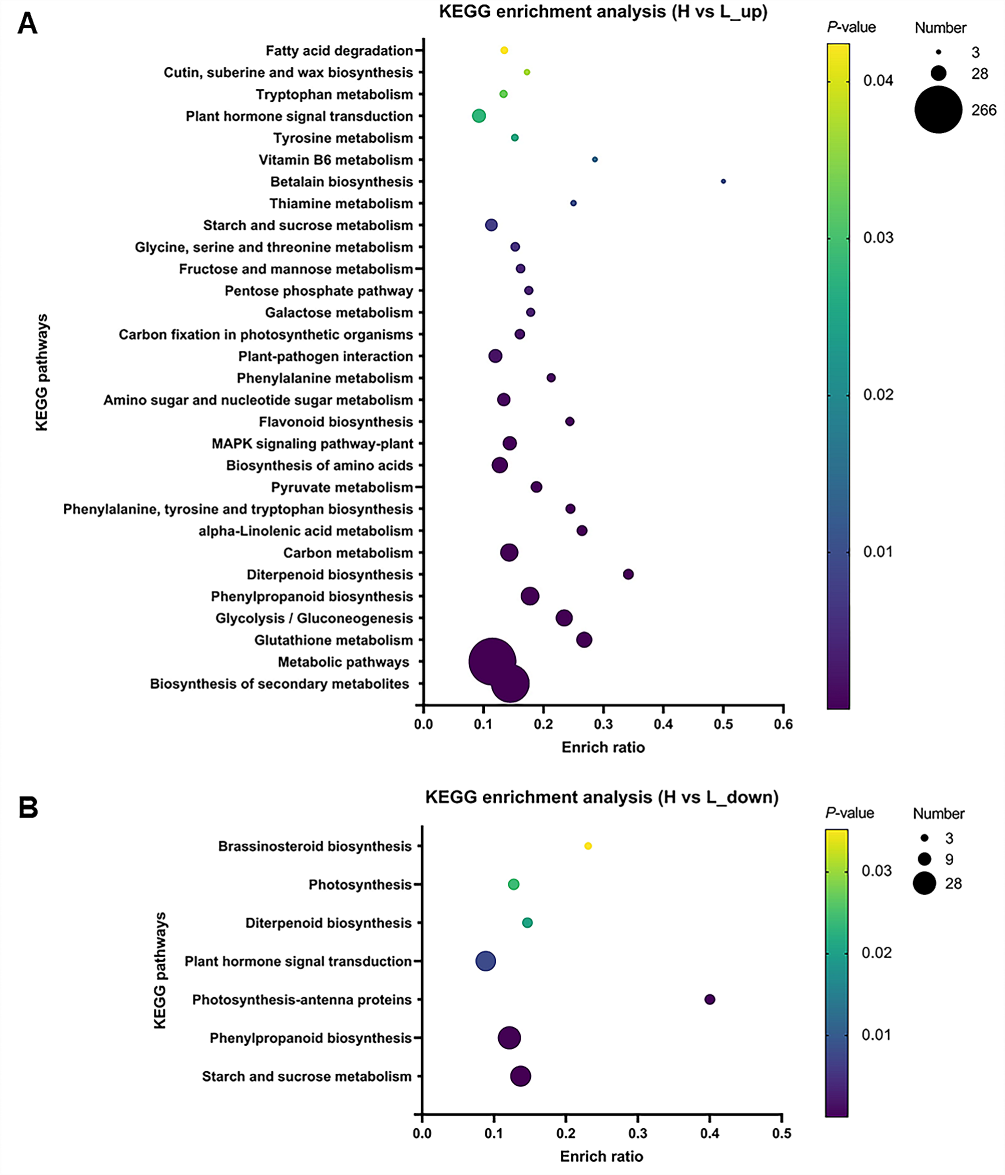
KEGG enrichment analysis. **A** KEGG enrichment analysis of genes that up-regulated expression in “H vs. L”. **B** KEGG enrichment analysis of genes that down-regulated expression in “H vs. L”. The vertical axis indicates KEGG pathways, and the horizontal axis indicates enrich ratio. The greater the enrich ratio, the greater the enrichment. The size of the dot indicates the number of genes in this KEGG pathway, and the color of the dot corresponds to different *p-adjust* ranges. Top30 enrichment results are displayed by default based on the premise of *p-adjust* < 0.05

The membrane transport and signal transduction were both related to environmental information processing in KEGG pathways (Additional file 1: Fig. S8). The plant hormone signal transduction was related to environmental information processing and was found in KEGG enrichment analysis of genes with both up-regulated and down-regulated expression in “H vs. L” (Fig. 5). Based on the phenotype of rhizome at different environment temperatures (Figs. 2–3) and GO and KEGG enrichment analysis, we speculated that plant hormones may play a major role in the response of the rhizome to temperature. It has been reported that auxin plays a major role in regulating axillary bud outgrowth, and it is also necessary for plant gravitropic responses (Li et al. 2020b; Liu et al. 2022b; Ongaro and Leyser 2007; Žádníková et al. 2015; Zhang et al. 2018). There were sixteen DEGs that associated with auxin-responsive genes in plant hormone signal transduction, including 5 *Aux*/*IAA* genes, 6 *GH3* genes and 5 *SAUR* genes. Except for 3 *Aux*/*IAA* genes *OsIAA7*, *OsIAA21* and *OsIAA25*, other 13 auxin-responsive genes were all down-regulated at low temperature (Additional file 1: Fig. S9), which indicated that auxin may play a significant role in rhizome development of *OL* in response to the high and low temperature.

**Fig. 6:**
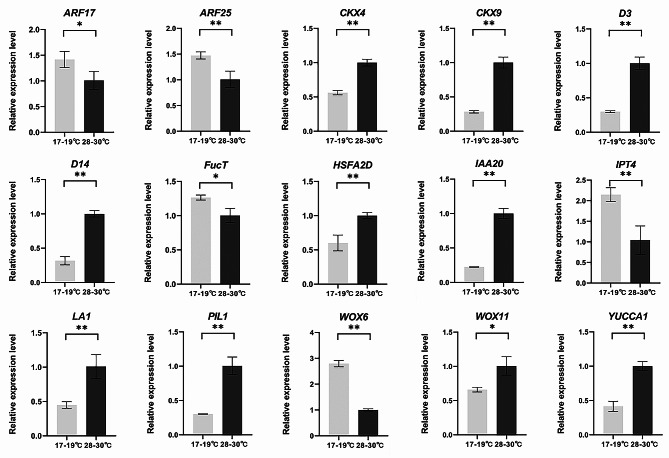
Relative expression levels of selected genes at about five days. The selected genes include *ARF17*, *ARF25*, *CKX4*, *CKX9*, *D3*, *D14*, *FucT*, *HSFA2D*, *IAA20*, *IPT4*, *LA1*, *PIL1*, *WOX6*, *WOX11* and *YUCCA1* between 17–19 ℃ and 28–30 ℃. Values are mean ± sd (*n* = 3). The statistical significance is determined by Student’s *t*-test; *, *P* < 0.05, **, *P* < 0.01

Based on GO and KEGG analysis, the DEGs (Additional file 2: Tables. S1-S2) that related to plant gravitropism (Harmoko et al. 2016; Li et al. 2020b; Zhang et al. 2018), sprouting of axillary buds (Duan et al. 2019; Gao et al. 2014; Liu et al. 2009b; Shibasaki et al. 2021; Yamamoto et al. 2007; Zhou et al. 2013) and transcription factors for plant temperature perception (Proveniers and van Zanten 2013; Todaka et al. 2012) were selected for quantitative real-time PCR (qPCR). The results showed that the upward and downward expression trend of these selected DEGs were consistent with that of transcriptome sequencing (Fig. 6; Additional file 1: Fig. S10). The expression levels of selected genes were further identified when the seedlings were cultured for about four and six days at 17–19 ℃ and 28–30 ℃, respectively. At about four days, the expression levels of *ARF17*, *ARF25*, *FucT*, *HSFA2D* and *YUCCA1* were not significantly different between 17–19 ℃ and 28–30 ℃ (Additional file 1: Fig. S11), and others were consistent with that at about five days (Fig. 6; Additional file 1: Fig. S11). At about six days, the expression levels of *ARF17*, *FucT* and *YUCCA1* were not significantly different between 17–19 ℃ and 28–30 ℃ (Additional file 1: Fig. S12), and others were consistent with that at about five days (Fig. 6; Additional file 1: Fig. S12). These results further confirmed the importance of selected genes in the response of *OL* to the temperature.

### Asymmetric Growth of Rhizome in Response to Different Temperature

Rhizomes were originated from the axillary buds of the crown and finally developed into an aerial stem (Fig. 1). In this process, rhizomes could keep underground growth for a period of time and show negative gravitropism. The asymmetric growth between the upper and the lower sides of plant organs in the response to gravity, leads to change of growth direction (Morita and Tasaka 2004; Vandenbrink and Kiss 2019). In *OL*, it has been reported that the upward growth of the rhizome is related to the asymmetric growth between the lower and upper sides of rhizome bent aera. When the rhizomes start to bend up, cells grow faster in the lower side of rhizome bent aera, resulting in the larger cell size than those in the upper side (Bessho-Uehara et al. 2018). Asymmetric distribution of auxin takes part in the gravitropic responses of rice, promoting the asymmetric expression of *IAA20*, *WOX6* and *WOX11* between the upper and the lower sides of shoot, which further leads to asymmetric growth (Li et al. 2020b; Zhang et al. 2018).

**Fig. 7:**
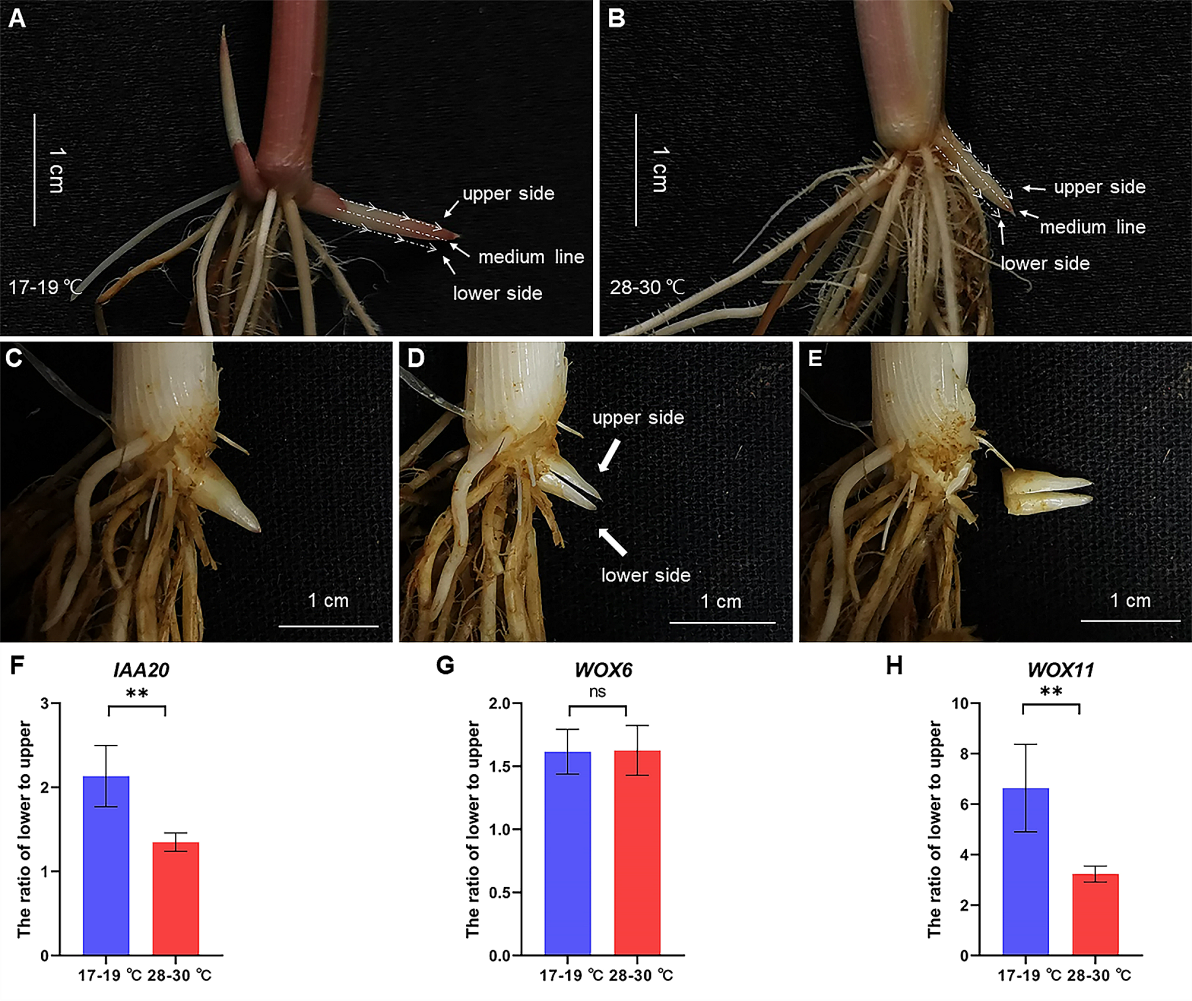
Expression of *IAA20*, *WOX6* and *WOX11* between upper and lower sides of rhizome (bud). **A** Rhizome of seedlings cultured at 17–19 ℃. **B** Rhizome of seedlings cultured at 28–30 ℃. **C** The rhizome (bud). **D** The rhizome (bud) is cut from the middle and divided into the upper and lower sides. **E** The rhizome (bud) separated from the mother plant. **F** The ratio of *IAA20* expression level between the upper and lower sides of the rhizome (bud). **G** The ratio of *WOX6* expression level between the upper and lower sides of the rhizome (bud). **H** The ratio of *WOX11* expression level between the upper and lower sides of the rhizome (bud). Values are mean ± sd (*n* = 9). The statistical significance is determined by Student’s *t*-test; ** *P* < 0.01, and ns represents no significance

Auxin-responsive marker gene *IAA20* and two transcription factor genes *WOX6* and *WOX11* (Zhang et al. 2018) for asymmetric growth were selected for analysis on the response of the rhizome to temperature. Our results showed that the upward growth of the rhizome was earlier at 17–19 ℃ than that at high temperatures (Fig. 3; Additional file 1: Fig. S6). Based on the time when rhizome (bud) began to bend up at 17–19 ℃ (about a week), the sampling time was divided into two stages (due to limited plant growth chambers): about 4-4.5 days (before the upward growth) and about 6-6.5 days (the upward growth began). The *OL* seedlings were cultured at 17–19 ℃ and 28–30 ℃, respectively (Fig. 7A-B). The rhizomes (buds) were cut longitudinally into the upper and the lower sides (Fig. 7C-E), and the two sides were collected for qPCR, respectively. The expression levels of *IAA20*, *WOX6* and *WOX11* in the lower side were all higher than that in the upper side (Additional file 1: Fig. S13), suggesting their involvement in the gravitropism. The ratio of *IAA20* expression level between the upper and lower sides was more obvious at 17–19 ℃ than that at 28–30 ℃ (Fig. 7F). At 17–19 ℃, the ratio of *WOX6* expression level between the lower and upper sides was not significantly different from that at 28–30 ℃ (Fig. 7G), indicating that *WOX6* was probably not involved in the temperature response. The ratio of *WOX11* expression level between the upper and lower sides was more obvious at 17–19 ℃ than that at 28–30 ℃ (Fig. 7H), suggesting that the extent of asymmetric growth between the upper and lower sides of the rhizome was larger at low temperature than that at high temperature. The above results indicated that aggravated asymmetric expression of auxin-responsive genes (*IAA20* and *WOX11*) and asymmetric growth may be related to earlier upward growth of the rhizome at lower temperature. The seedlings were cultured at 17–19 ℃, and the rhizome (bud) began bending up at about 6-6.5 days (Additional file 1: Fig. S14A). The expression level of *IAA20* in the lower side was not significantly different from that in the upper side (Additional file 1: Fig. S14B), while the expression differences of *WOX6* and *WOX11* between the upper and the lower sides were just like that before the upward growth began (Additional file 1: Fig. S14C-E). When the rhizome (bud) began bending up, the ratio of *WOX6* expression between the upper and lower sides was not significantly different at 17–19 ℃ and 28–30 ℃ (Additional file 1: Fig. S14F). The ratio of *WOX11* expression between the upper and lower sides was still more obvious at 17–19 ℃ than that at 28–30 ℃ (Additional file 1: Fig. S14G). These results further suggested that compared with higher temperature, lower temperature could promote negative gravitropic response of the rhizome, resulting in earlier upward growth (asymmetric growth) of *OL* rhizome.

## Discussion

Human activities have intensified climate change, which has brought negative impacts on sustainable agricultural development (Anderson et al. 2016; Coumou and Rahmstorf 2012; Fuglie 2021). Rhizomatous *OL* has strong adaptability to the environment and the same AA-type genome as cultivated rice, making it an ideal genetic resource for developing perennial rice (Hu et al. 2003; Tao and Sripichitt 2000; Zhang et al. 2023). The rhizome originating from the axillary bud at the shoot base is a kind of underground stem that can grow underground like roots and eventually develop into new aerial stems (Gizmawy et al. 1985). Plant gravitropism refers to the reorientation of plant growth under the action of gravity, which is an important environmental factor determining plant morphology (Bastien et al. 2014). The gravitropism of the rhizomes is related to the developmental process of rhizomes from buds into new aerial stems (Fig. 1C). The cells in the lower side of the rhizome grow faster than that in the upper side, when the rhizome starts to bend up (Bessho-Uehara et al. 2018). The later of the rhizome developing into aerial stems, the longer of the rhizome, while the earlier of the rhizome developing into aerial stems, the shorter of the rhizome (Fig. 1C). Fine temperature control experiments in the plant chamber showed that the upward growth of the rhizome (bud) was earlier at 17–19 ℃ than that at 28–30 ℃, resulting in shorter rhizomes at 17–19 ℃ than that at 28–30 ℃, and the branch angle was also smaller than that at 28–30 ℃ (Fig. 3; Additional file 1: Fig. S6).

**Fig. 8:**
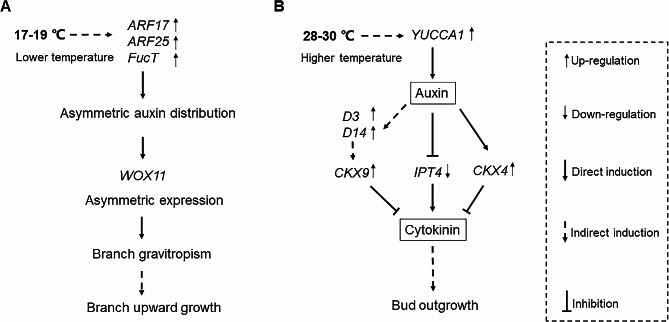
The model for the effect of temperature on gravitropism and outgrowth of branch. **A** Compared with 28–30 ℃, the effect of lower temperature on branch gravitropism. **B** Compared with 17–19 ℃, the effect of higher temperature on controlling outgrowth of branch

How shoot gravitropism of rice act in response to different temperatures is not clear now (Wang et al. 2022). Our results showed that the response of the rhizome to the gravity at the low and the high environment temperature was different. The low temperature could enhance the negative gravitropism of the rhizome, while high temperature could attenuate the gravitropic response (Fig. 3; Additional file 1: Fig. S6). The formation of asymmetric auxin distribution will lead to asymmetric growth in the upper and lower sides of responding organs to gravity (Hashiguchi et al. 2013). The significant expression difference of the auxin-responsive marker gene *IAA20* between the upper and lower sides of the rhizome at 17–19 ℃ and 28–30 ℃ (Fig. 7F) showed that asymmetric auxin distribution may take part in regulating upward growth of the rhizome. *LA1-*dependent gravity signaling pathways play an important role in rice shoot gravitropism. *LA1* promotes the shoot gravitropism through asymmetric redistribution of auxin and reducing the expression of *LA1* or loss of *LA1* function will lead to reduced gravitropism and larger tiller angle phenotypes (Yoshihara and Iino 2007; Zhang et al. 2018). Heat shock transcription factor (*HSFA2D*) can be induced by the high temperature (Liu et al. 2009a). *HSFA2D* acts upstream of *LA1* and positively regulates the expression of *LA1* in rice shoot gravitropism, which further regulates the asymmetric expression of *WOX6* and *WOX11* (Hu et al. 2020; Zhang et al. 2018). In our results, the expression of *HSFA2D* and *LA1* were up-regulated at 28–30 ℃ and down-regulated at 17–19 ℃ (Fig. 6), but the branch angle was larger at 28–30 ℃ (Fig. 3; Additional file 1: Fig. S6), which suggested that there might be *LA1*-independent pathways that led to asymmetric redistribution of auxin. In rice, multiple *LA1*-independent pathways and genes regulating the response to gravity can also play important role in determining plant morphology (Harmoko et al. 2016; Li et al. 2020b, 2021). Knocking out of *OsARF12*, *OsARF17* and *OsARF25* can lead to a larger tiller angle, suggesting that *LA1*-independent pathways are involved (Li et al. 2020b). The gene *FucT*, as α 1,3-fucosyltransferase, can affect the basipetal auxin transport at the shoot base of rice, and loss function of it attenuates gravitropic response, which leads to increased tiller angle (Harmoko et al. 2016). The expression of *LA1* was not significantly different between wild-types and *fuct-1* mutants with loss of *FucT* function (Harmoko et al. 2016). In our results, the expression of *ARF17*, *ARF25* and *FucT* were all up-regulated at 17–19 ℃ (Fig. 6). Compared with 28–30 ℃, the expression difference of *WOX11* between the upper and lower sides of rhizome was significantly enhanced at 17–19 ℃ (Fig. 7H). Based on our results, we proposed a model for the rhizome (bud) gravitropism at lower temperature (Fig. 8A). Compared with that at higher temperature, the expression levels of *ARF17*, *ARF25* and *FucT* were up-regulated at 17–19 ℃, resulting in prospectively asymmetric auxin distribution between the upper and lower sides of the branch. Asymmetric auxin distribution further led to asymmetric expression of *WOX11*, resulting in asymmetric growth between the upper and lower sides of branch, so that the upward growth of the branch at lower temperature was earlier than that at higher temperature.

The gene *OsPIL1* (also named *OsPIL13*) is a transcription factor responding to temperature stimulus, and its expression level is increased by high temperature (Proveniers and van Zanten 2013; Todaka et al. 2012). The expression of *OsPIL1* was down-regulated at 17–19 ℃ (Fig. 6), which suggested that the plant growth had already perceived the difference of environment temperature. Lower temperature (< 20 °C) will inhibit tillering of the cultivated rice at vegetative stage (Sánchez et al. 2014; Shimono et al. 2002), while the axillary bud in the shoot base of *OL* seedlings began sprouting earlier at 17–19 ℃ (Fig. 2), suggesting that *OL* had stronger ability to adapt to the lower temperature than cultivated rice.

The sprouting of buds in plants is controlled by multiple hormones. Auxin, cytokinin and strigolactone play a major role in regulating the shoot branch (Kotov et al. 2021; Rameau et al. 2015). Auxin negatively regulating the outgrowth of bud, is the first hormone identified in apical dominance (Rameau et al. 2015). High temperatures can promote auxin biosynthesis, while low temperatures can decrease the auxin level in *Arabidopsis* (Gray et al. 1998). *YUCCA1* is an important enzyme for auxin biosynthesis in rice (Yamamoto et al. 2007). The *YUCCA1* and auxin response marker gene *IAA20* were up-regulated at 28–30 ℃ and down-regulated at 17–19 ℃, which suggested that the level of auxin may be higher at high temperature than that at low temperature in *OL.* Auxin can control cytokinin biosynthesis by inhibiting the expression of *IPT4* (Zhang et al. 2010). The expression of *IPT4* was induced by nitrogen supply, which positively regulates cytokinin biosynthesis, promoting *OL* rhizome bud outgrowth (Shibasaki et al. 2021). The *CKX4* that belongs to *CYTOKININ OXIDASE*/*DEHYDROGENASE* (*CKX*) can decrease the level of cytokinin, suppressing outgrowth of bud (Wang et al. 2021), and the expression level of *CKX4* can be increased by auxin (Gao et al. 2014). The down-regulation of the *IPT4* and up-regulation of the *CKX4* at 28–30 ℃ (Fig. 6) suggested that high temperature could induce lower cytokinin levels in the shoot base and would lead to the dormancy of axillary buds. Strigolactone is a new phytohormone, that negatively regulates bud outgrowth. The signal transduction of strigolactone is affected by auxin. The expression of *D3* and *D14* were increased when treated with GR24 (a synthetic strigolactones), NAA (α-naphthylacetic acid) and the combined NAA and GR24 in tall fescue, while decreased with NPA (auxin transport inhibitor N-1-naphthylphalamic acid) or the combined NPA and GR24 treatment (Hu et al. 2018, 2019). The *D3* gene is strongly expressed in the shoot base, and D3 is an F-box protein with rich leucine repeats that inhibits the activity of rice tillering buds and maintains bud dormancy (Ishikawa et al. 2005; Zhao et al. 2014). The *D14* gene one of strigolactone signal transduction, negatively regulates tiller bud outgrowth in rice, and reducing the expression of *D14* can lead to more tillers (Liu et al. 2009b). Strigolactone induces degradation of the D53 protein, a repressor of strigolactone signaling in rice, through D14–SCF^D3^ in strigolactone signaling pathways (Jiang et al. 2013; Zhou et al. 2013) and further enhances the expression of *CKX9* that belong to *CYTOKININ OXIDASE*/*DEHYDROGENASE* (*CKX*) to decrease the level of cytokinin in rice (Duan et al. 2019). The functions of *CKX4* and *CKX9* are overlapping in rice. Compared with wild type, the level of cytokinin is increased in *CKX4* and *CKX9* double mutant, and the *CKX4* and *CKX9* double mutant has a significantly larger number of tillers (Rong et al. 2022). The expression of *D3*, *D14* and *CKX9*, which negatively regulate bud outgrowth in strigolactone pathways, were all up-regulated at 28–30 ℃ (Fig. 6). These results suggested that, apart from auxin and cytokinin, strigolactones pathways were also involved in controlling the sprouting of axillary buds at different temperatures. Based on our transcriptome sequencing and qPCR of differently expressed genes, we proposed a possible model for the effect of higher temperature on the sprouting of axillary buds (Fig. 8B). In this model, the expression of auxin biosynthesis gene *YUCCA1* was upregulated, resulting in a higher auxin level at higher temperature. Auxin negatively regulated the cytokinin level by inhibiting the expression of cytokinin biosynthesis gene *IPT4* and promoting the expression of cytokinin oxidase/dehydrogenase genes *CKX4* (Gao et al. 2014; Zhang et al. 2010). The *D3* and *D14* in strigolactone signal transduction pathways were up-regulated by auxin, and further promoting the expression of cytokinin oxidase/dehydrogenase genes *CKX9*, which also negatively regulated the cytokinin level (Duan et al. 2019; Hu et al. 2018, 2019; Jiang et al. 2013; Zhou et al. 2013). The level of cytokinin was down-regulated by auxin and strigolactone, resulting in the delayed outgrowth of axillary bud at higher temperature.

*OL* with a stronger ability to adapt to environments, is an important genetic resource for the improvement of cultivated rice, and the research of rhizome will be meaningful for breeding perennial rice. *OL* is originated from the area of Africa with savanna climate that is characterized by year-round high temperatures, distinct dry and wet seasons. There is almost no rainfall during the dry season, which is not suitable for plant growth, and local agricultural production heavily relies on rainfall at wet season (Mechiche-Alami and Abdi 2020). Usually, rainfall is accompanied by a certain degree of temperature decrease, and the growth habit of *OL* at lower temperature can help plants quickly cover the land surface and occupy ecological niche when the wet season begins, which has a positive effect on ecological restoration.

## Conclusion

In this study, fine temperature treatment of *OL* proved that the outgrowth of axillary buds and rhizome gravitropism were greatly influenced by environment temperature. The results of comparative transcriptome showed that plant hormones and plant hormone signal transduction played an essential role in the response of *OL* to temperature. Compared with higher temperature, lower temperature could enhance negative gravitropism of the rhizome (bud), so that upward growth of the rhizome (bud) was earlier than that at higher temperature. Compared with higher temperature, lower temperature could also promote the sprouting of the axillary bud.

## Materials and methods

### Plant Materials and Environmental Conditions

*OL* introduced from Africa, was planted in the experimental field at Guangxi University and bagged for harvesting seeds from 2017 to 2022. The fine temperature control experiment was carried out in the plant growth chamber (PERCIVAL USA E-41L1). The seedlings were used for different temperature treatments when the seeds germinated and grew to the 4–5 leaf stage (Additional file 1: Fig. S15). Hydroponics was used for selecting the range of temperature, and the formula of the hydroponic solution was shown in Additional file 2: Table S3. Five environmental temperature ranges including 17–19 ℃, 20–22 ℃, 25–27 ℃, 28–30 ℃ and 30–32 ℃ were selected for analyzing the influence of different temperatures to the rhizome development. Seedlings were cultured at 12 h of light and 12 h of darkness, and the humidity was set at 65%. To simulate the natural environment of rice growth, based on the results of hydroponics, we planted *OL* seedlings in rice paddy soil with a low temperature of 17–19 ℃ and a high temperature of 28–30 ℃.

### Phenotypic Statistics of Rhizome

The fine temperature control experiments of *OL* seedlings were carried out in the plant growth chamber. The angle between axillary bud and mother plant was measured after 5–10 days of simultaneous cultivation in the plant growth chamber at different temperatures. The rhizome lengths and the angle between the branch and the mother plant of *OL* were measured after about three-four weeks.

### Transcriptome Sequencing of Crowns at low and high Environmental Temperature

To reduce the impact of bud sprouting speed at different temperatures, seedlings were cultured at 26 ℃ and then cultured at 17–19 ℃ and 28–30 ℃ in the plant growth chamber when the first axillary bud of the shoot base began to sprout. During this period, some seedlings were dug out from the soil to observe the development of axillary buds at the shoot base. For about five days, seedlings dug out from the soil were cleaned with water, and the crowns of seedlings were harvested and frozen immediately in liquid nitrogen (Additional file 1: Fig. S16) when the angle between axillary bud and seedlings would be about to emerge at the low and high temperature. Each temperature group contained three biological repeats, and the crowns from at least 20 seedlings were put together to form a biological repeat. Transcriptome sequencing was entrusted to Majorbio (Shanghai China) and carried out at Illumina Novaseq 6000 platform. The data were analyzed on the online platform of Majorbio Cloud Platform (www.majorbio.com).

### Identification of DEGs

Analysis of transcriptome sequencing was according to *Oryza sativa*. Reference Genome Version is IRGSP-1.0 (http://plants.ensembl.org/Oryza_sativa/Info/Index). The quantitative analysis of gene level was performed using the expression quantitative software RSEM, with TPM (Transcripts Per Kilobase Million) as an indicator. After obtaining the read counts of genes through gene expression analysis, DESeq2 software was used to analyze the differential expression of genes between groups for multi samples with default parameters: *P*-*adjust* < 0.05 and |log_2_FC| ≥ 1. The up-regulated and down-regulated genes in “H vs. L”, were selected for KEGG and GO enrichment analysis, respectively. The GO enrichment analysis was carried out in g: Profiler (Kolberg et al. 2023). The KEGG enrichment analysis was carried out in KOBAS (Bu et al. 2021).

### Quantitative Real-Time PCR

Fifteen differently expressed genes were selected for quantitative real-time PCR. Seedlings cultured at 17–19 ℃ and 28–30 ℃, and the crowns of seedlings were harvested for qPCR at about four and six days. Each sample contained three biological repeats, and 10–20 seedlings were pooled for each biological repeat. At 17–19 ℃ and 28–30 ℃, the upper and lower side of the rhizome (bud) were collected for qPCR at about four and half days (before bending up) and six and half days (bending up was started). Each sample contained three biological repeats, and at least 15 seedlings were pooled for each biological repeat. All samples were pestled with liquid nitrogen and put in trizol (Trans Gen Biotech Beijing China). RNA was extracted under RNase-free conditions via extraction of total RNA from rice tissues (Fang et al. 2018). Reverse transcription reactions were performed with HiScript II 1st Strand cDNA Synthesis Kit (+ gDNA wiper) (Vazyme Nanjing China). Quantitative real-time PCR was performed using ChamQ Universal SYBR qPCR Master Mix (Vazyme Nanjing China) on a real-time system (Light Cycler 480). Three biological replicates were performed for each gene and three technical replicates for each biological replicate. The rice *Ubiquitin* gene (*LOC_Os03g13170*) was used as an internal control, and expression level were calculated according to the 2^(−ΔΔCt)^ analysis method (Livak and Schmittgen 2001). Analysis of transcriptome sequencing was according to *Oryza sativa*, and the primers used for qPCR were obtained from the sequence of *O. sativa* genome. The primer sequence should be aligned with the sequence of *OL* genome. The sequence of *OL* genome has been reported in previous studies (Li et al. 2020a; Reuscher et al. 2018). We used the “BLAST” to determine whether the primers (obtained from the sequence of *O. sativa* genome) were consistent with the sequence of *OL* genome (Reuscher et al. 2018), websites: http://133.39.75.173/?page_id=9. The gene-specific primers used for qPCR the were listed in Additional file 2: Table S4.

### Electronic Supplementary Material

Below is the link to the electronic supplementary material.

Supplementary Material 1

Supplementary Material 2

## Data Availability

All data supporting the findings of this study are available from the corresponding author on reasonable request.

## Abbreviations

*OL*: *Oryza longistaminata*
g: Gravity
H: High temperature (28–30 ℃)
L: Low temperature (17–19 ℃)
*CKX*: *CYTOKININ OXIDASE*/*DEHYDROGENASE*
Quantitative real-time PCR: qPCR
GR24: A synthetic strigolactones
NAA: α-naphthylacetic acid
NPA: Auxin transport inhibitor N-1-naphthylphalamic acid
