# Supplementary material for: Temperature Effect on Rhizome Development in Perennial rice
Source: Rice (N Y). 2024 May 8;17:32. doi: 10.1186/s12284-024-00710-2 (PMC11078906; doi:10.1186/s12284-024-00710-2)
Supplement: Supplementary file 1 — Supplementary Material 1 [file 12284_2024_710_MOESM1_ESM.docx]

**Additional Material**

**Temperature effect on rhizome development in perennial rice**

Kai Wang, Jie Li, Yourong Fan^*^, Jiangyi Yang^*^

^*^ Correspondence: [yangjy598@163.com](mailto:yangjy598@163.com); [fanyourred@163.com](mailto:fanyourred@163.com)

State Key Laboratory for Conservation and Utilization of Subtropical Agro-Bioresources, College of Life Science and Technology, Guangxi University, Nanning 530004, China


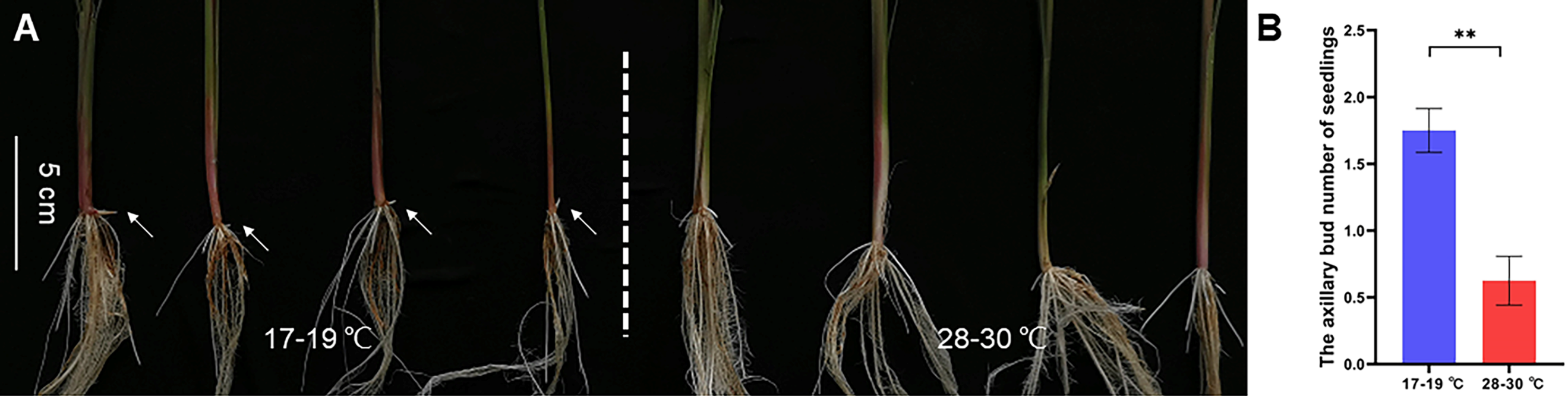


**Fig. S1** The axillary bud outgrowth of *OL* seedlings in hydroponics at 17-19 ℃ and 28-30 ℃. **A** The seedlings cultured in hydroponics at 17-19 ℃ and 28-30 ℃ for 10 days. **B** The average number of sprouted axillary buds per seedling at 17-19 ℃ and 28-30 ℃. Values are mean ± se (*n* = 9). The statistical significance is determined by Student's *t*-test; **, *P* < 0.01. The white arrows represent new branches (tillers or rhizomes).


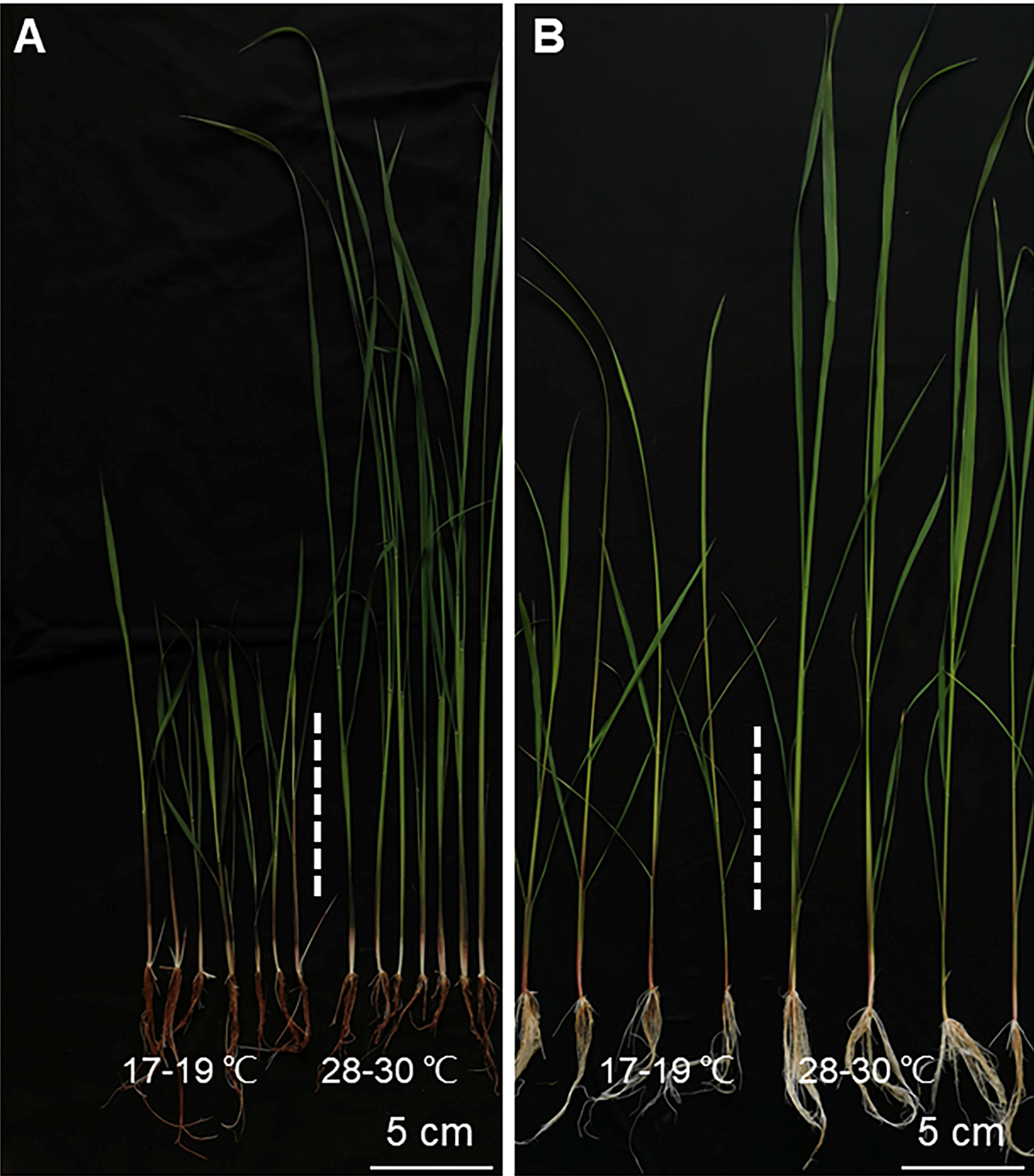


**Fig. S2** The seedlings cultured at 17-19 ℃ and 28-30 ℃ in plant growth chamber. **A** The seedlings cultured in the rice paddy soil. **B** The seedlings cultured in the hydroponics.


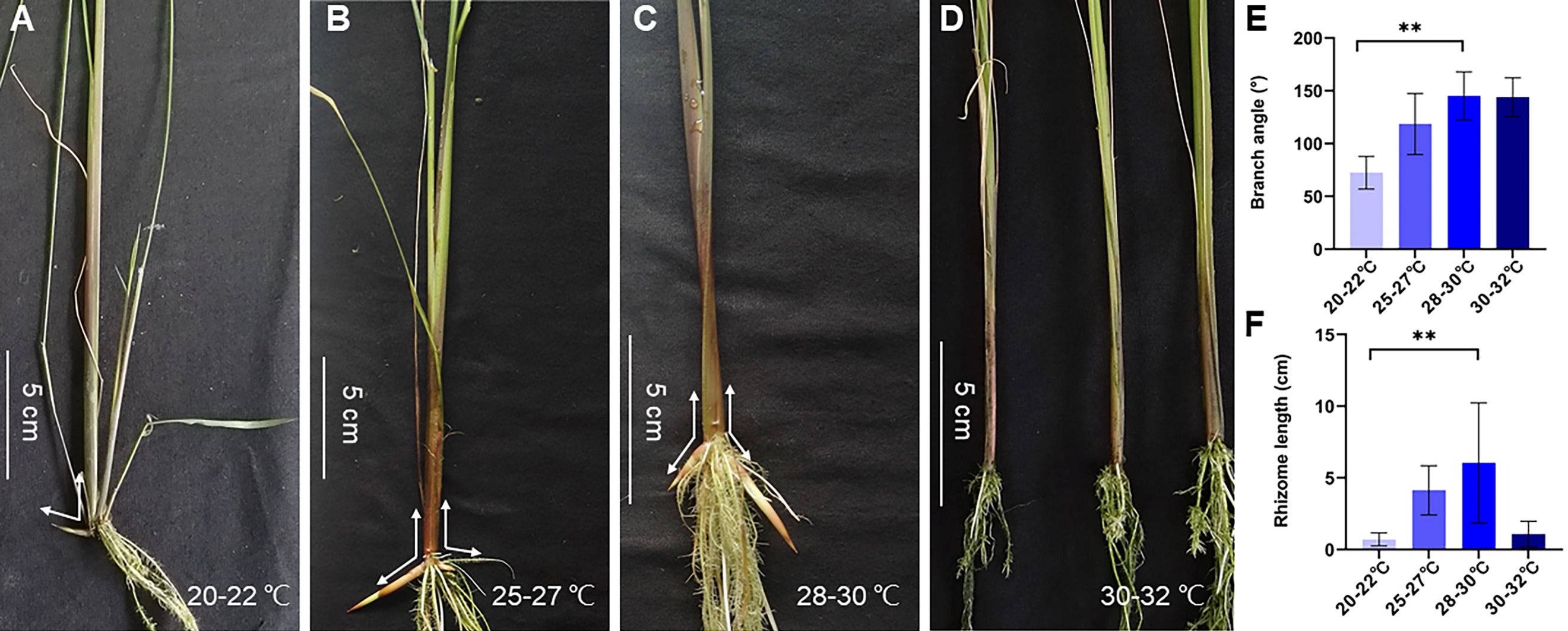


**Fig. S3** The shoot branch in hydroponics at different temperatures. **A** The seedlings cultured at 20-22 ℃. **B** The seedlings cultured at 25-27 ℃. **C** The seedlings cultured at 28-30 ℃. **D** The seedlings cultured at 30-32 ℃. **E** The difference of branch angle at four temperatures. **F** The difference of rhizome length at four temperatures. Values are mean ± sd (*n* = 30). The statistical significance is determined by Student's *t*-test, ** *P* < 0.01.


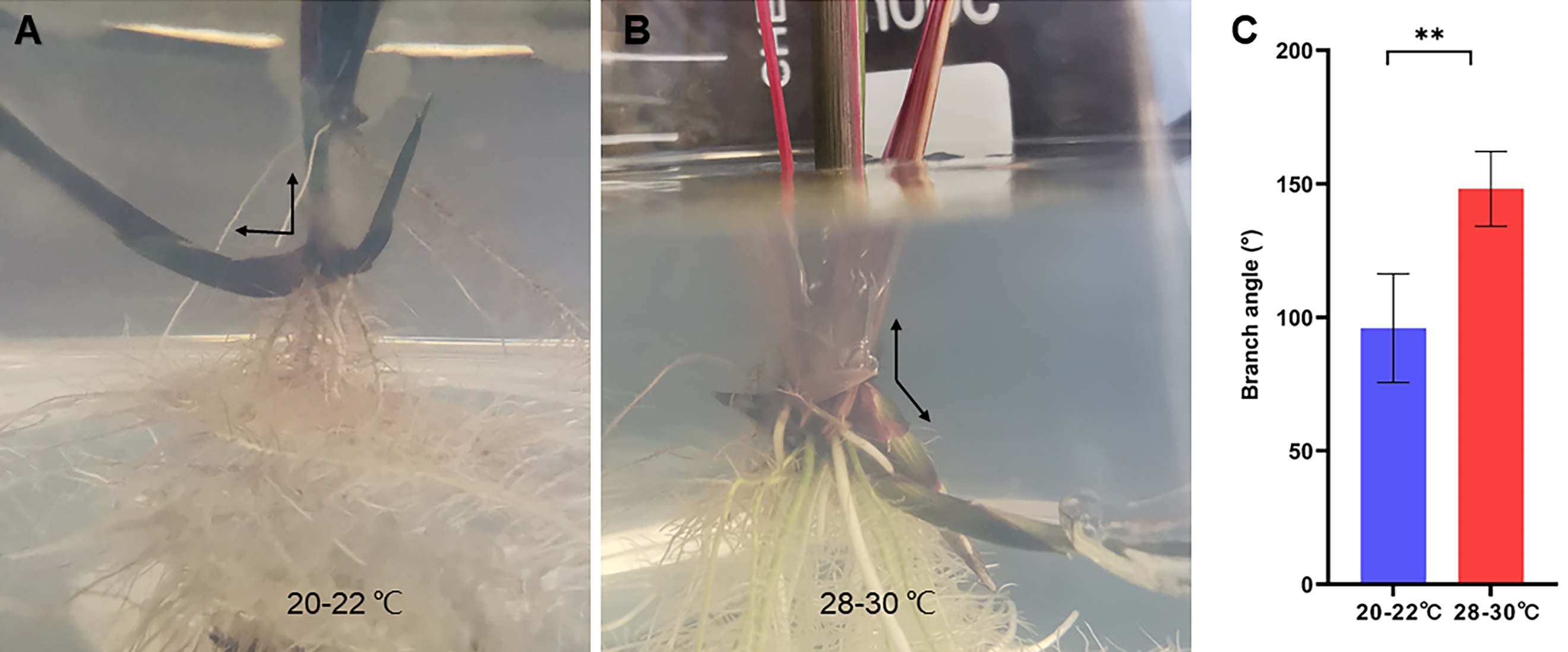


**Fig. S4** The growth difference of rhizome cultured in rooting medium at 20-22 ℃ and 28-30 ℃. A Seedings at 20-22 ℃, B Seedings at 28-30 ℃. C The difference of branch angle at 20-22 ℃ and 28-30 ℃. Values are mean ± sd (*n* = 9). The statistical significance is determined by Student's *t*-test, ** *P* < 0.01.


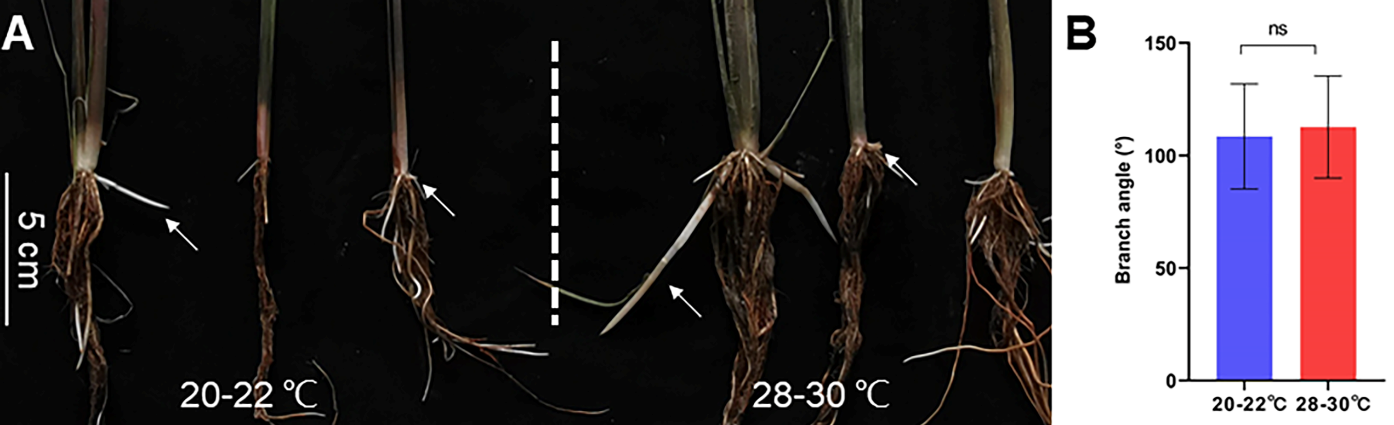


**Fig. S5** The growth of rhizome cultured in rice paddy soil at 20-22 ℃ and 28-30 ℃. **A** Seedlings are cultured at 20-22 ℃ and 28-30 ℃. **B** Branch angle of seedings at 20-22 ℃ and 28-30 ℃. Values are mean ± sd (*n* = 20). The statistical significance is determined by Student's *t*-test, and ns represents no significance. The white arrows represent new branches (tillers or rhizomes).


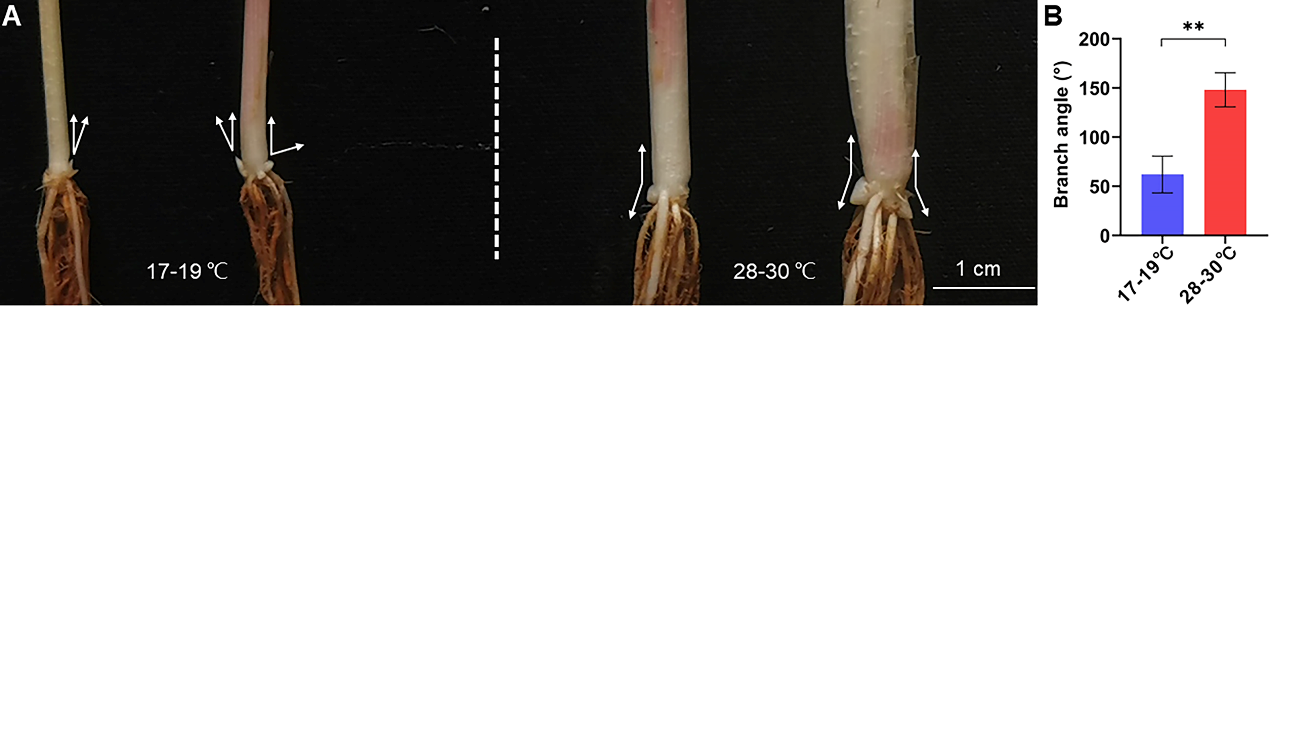


**Fig. S6** The branch angle of *OL* seedlings planted at 17-19 ℃ and 28-30 ℃. **A** The axillary bud of seedlings at 17-19 ℃ and 28-30 ℃. **B** The average branch angle at 17-19 ℃ and 28-30 ℃. Values are mean ± sd (*n* = 30). The statistical significance is determined by Student's *t*-test; **, *P* < 0.01. The white arrow represents the angle between the branch and mother plant.


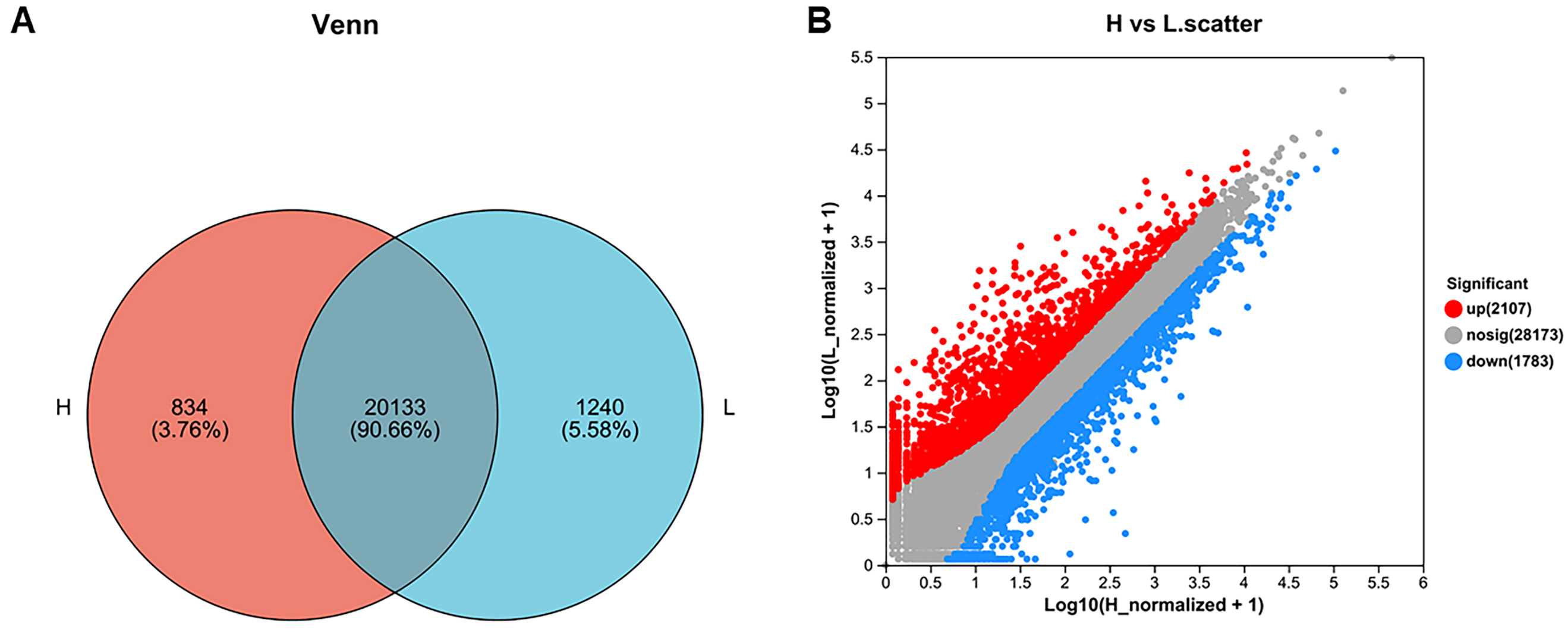


**Fig.** **S7** The Venn and scatter plot of DEGs in the transcriptome analysis. **A** Venn plot analysis of the “H vs L”. **B** Scatter plot of DEGs of the “H vs L”. The red indicates up-regulation, the light blue represents down-regulation, and the grey represents noise (no difference). The “H” represents the high temperature (28-30 ℃), and the “L” represents the low temperature (17-19 ℃).


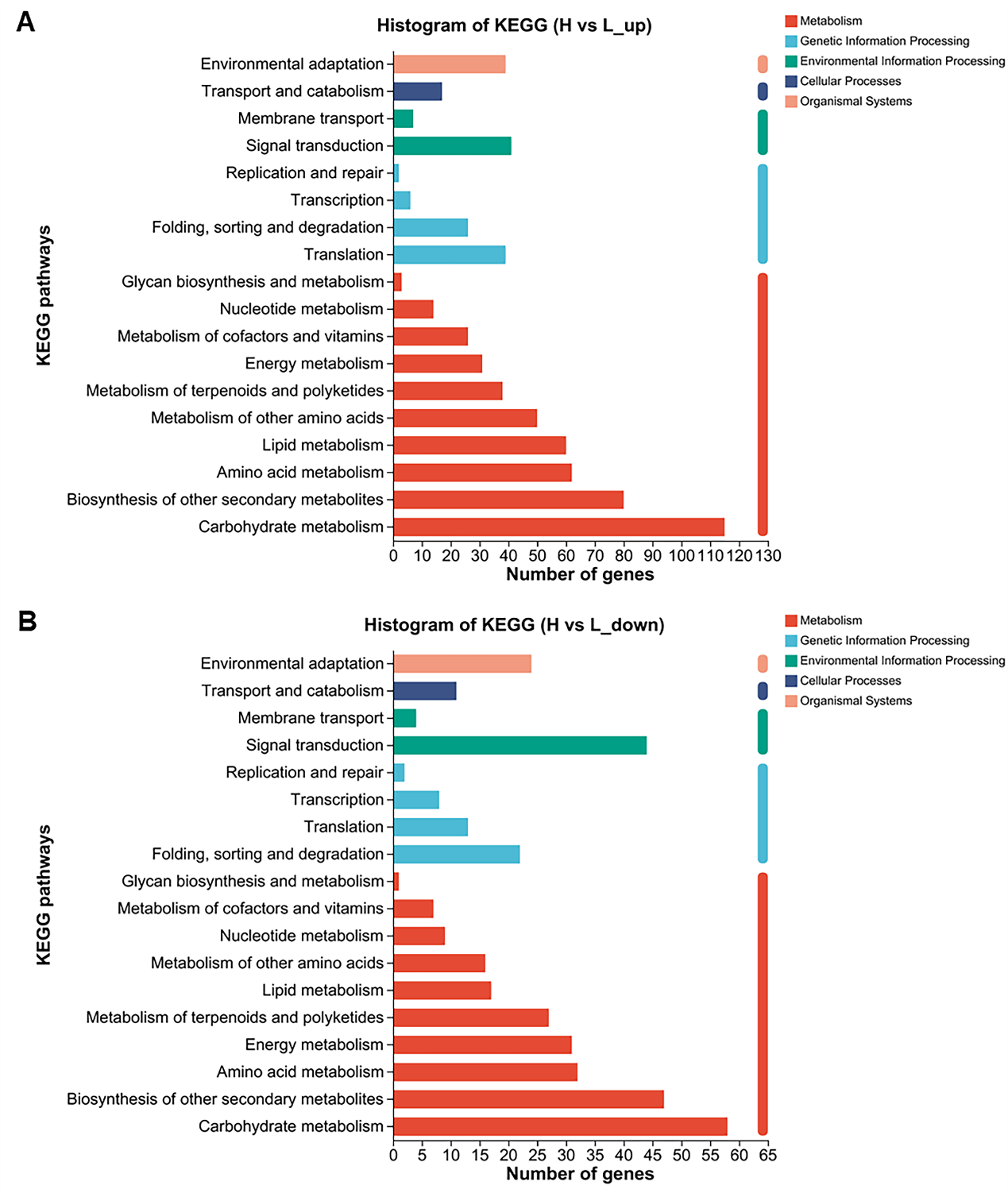


**Fig. S8** The histogram of KEGG. **A** The KEGG histogram of genes that up-regulated expression in “H vs L”. **B** The KEGG histogram of genes that down-regulated expression in “H vs L”. The ordinate represents the KEGG pathways, and the horizontal axis represents the number of genes. Different colors indicated different branches of the KEGG metabolic pathways.


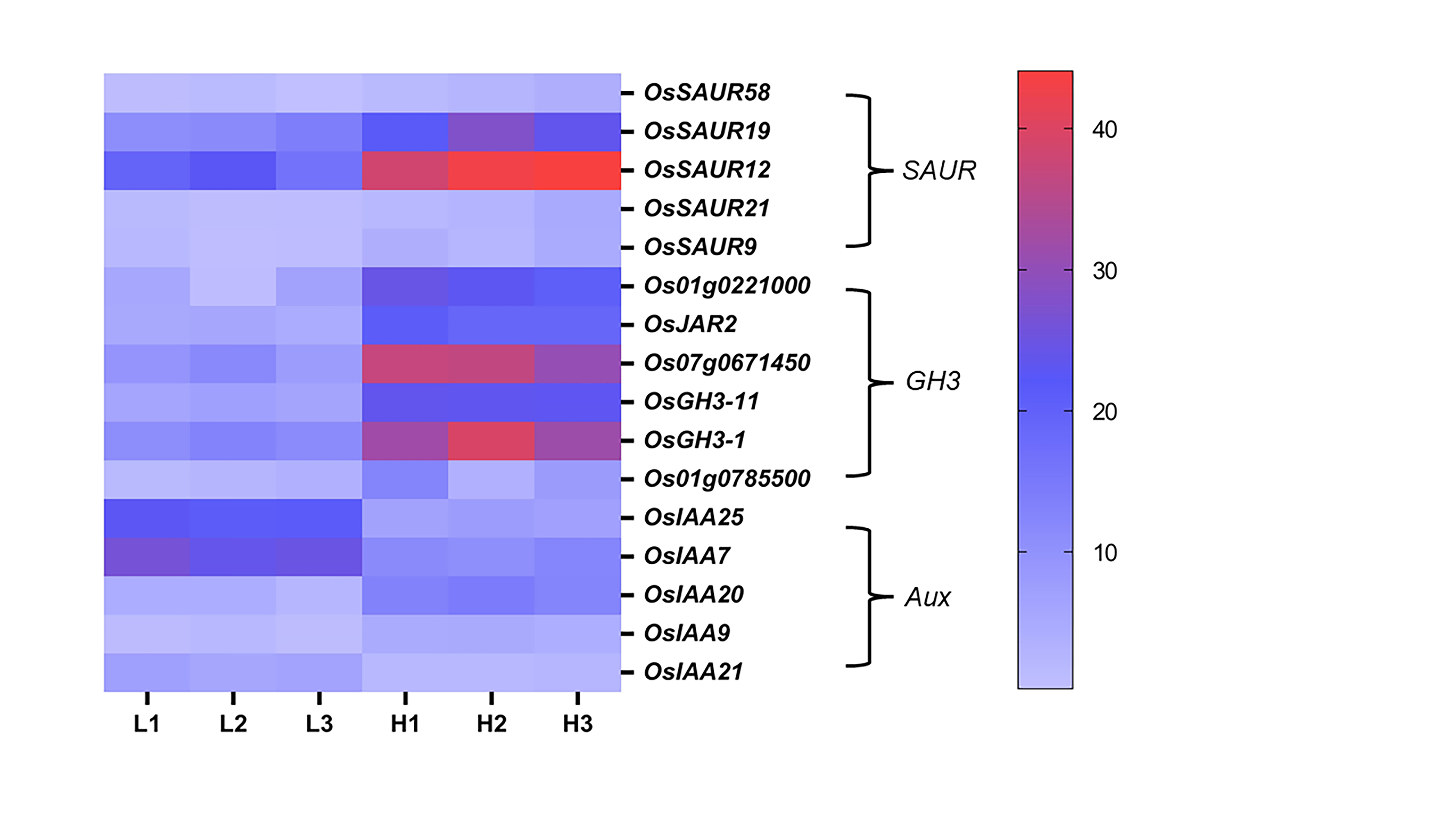


**Fig.** **S9** Heat map of auxin-responsive genes expression in KEGG enrichment analysis. L represents 17-19 ℃, H represents 28-30 ℃. Each column in the figure represents a sample, and each row represents a gene. The color in the figure represents the expression level of the gene in each sample. The red represents a higher expression level of the gene in the sample, and the blue represents a lower expression level.


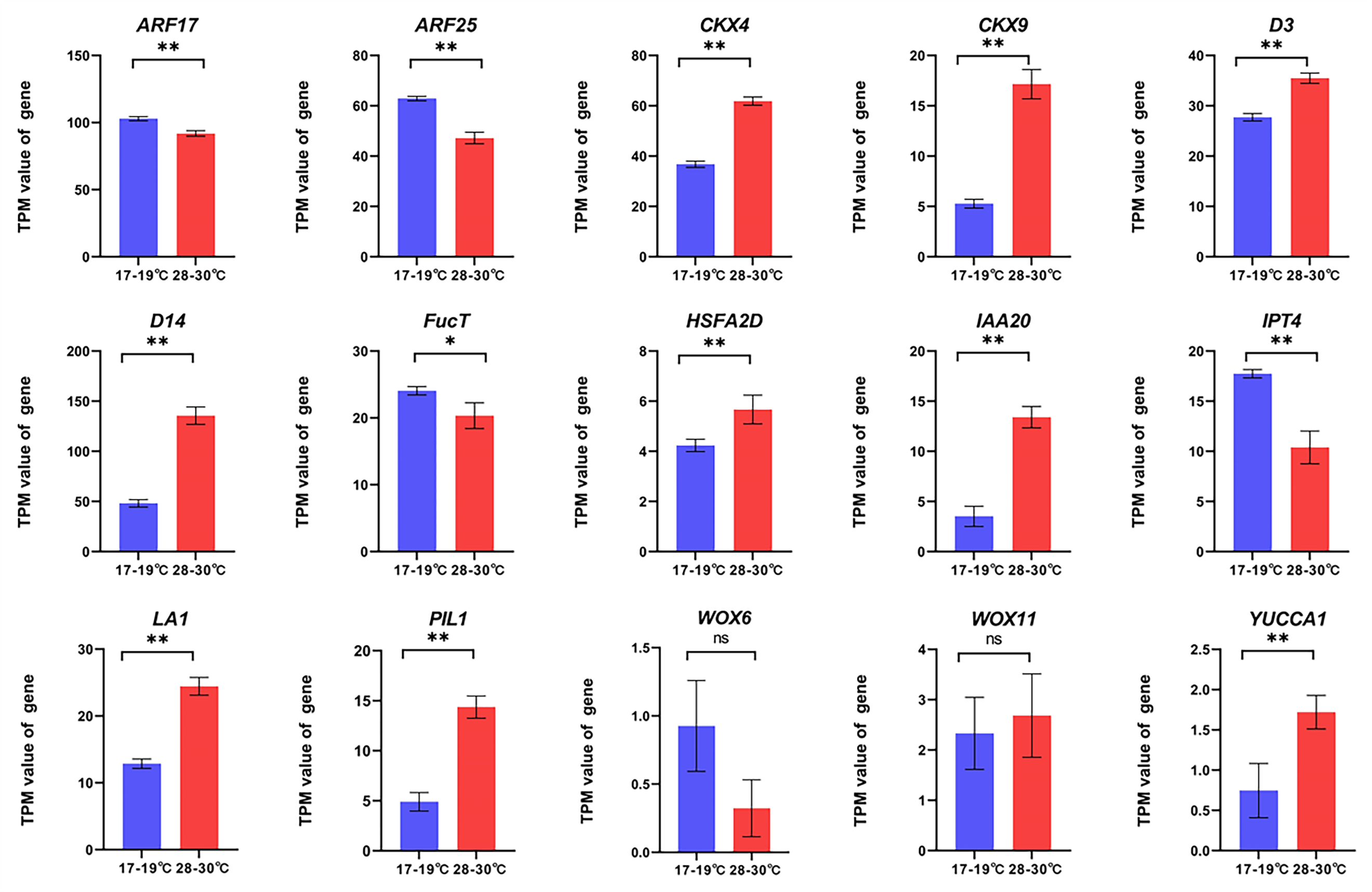


**Fig.** **S10** TPM (Transcripts Per Kilobase Million) value of selected genes at 17-19 ℃ and 28-30 ℃. The selected genes include *ARF17*, *ARF25*, *CKX4*, *CKX9*, *D3*, *D14*, *FucT*, *HSFA2D*, *IAA20*, *IPT4*, *LA1*, *PIL1*, *WOX6*, *WOX11* and *YUCCA1*. Values are mean ± sd (*n* = 3). The statistical significance is determined by Student's *t*-test; *, *P* < 0.05, **, *P* < 0.01, and ns represents no significance.

**Fig. S11** Relative expression levels of selected genes at about four days. The selected genes include *ARF17*, *ARF25*, *CKX4*, *CKX9*, *D3*, *D14*, *FucT*, *HSFA2D*, *IAA20*, *IPT4*, *LA1*, *PIL1*, *WOX6*, *WOX11* and *YUCCA1* between 17-19 ℃ and 28-30 ℃. Values are mean ± sd (*n* = 3). The statistical significance is determined by Student's *t*-test; *, *P* < 0.05, **, *P* < 0.01, and ns represents no significance.


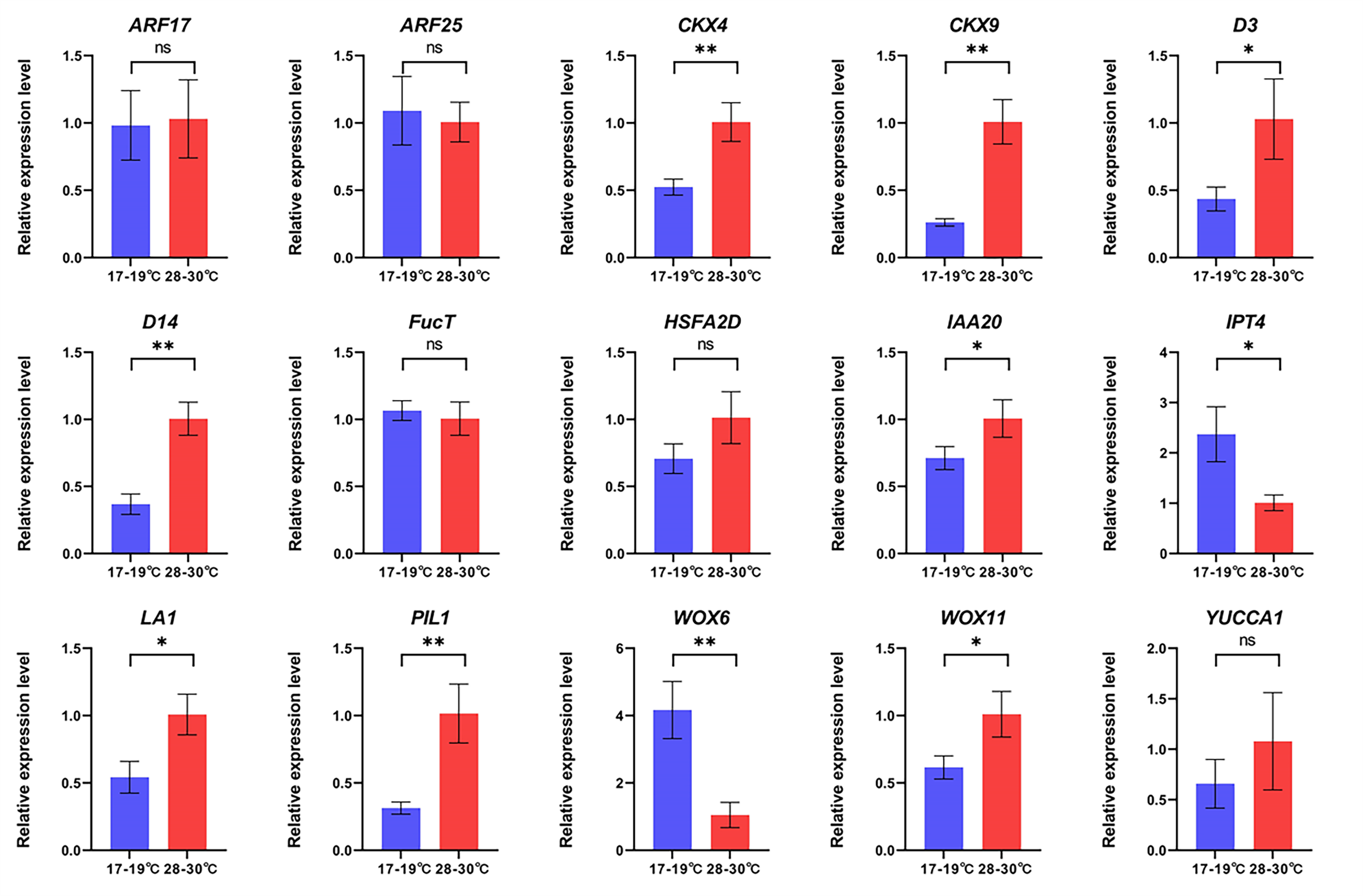


**Fig. S12** Relative expression levels of selected genes at about six days. The selected genes include *ARF17*, *ARF25*, *CKX4*, *CKX9*, *D3*, *D14*, *FucT*, *HSFA2D*, *IAA20*, *IPT4*, *LA1*, *PIL1*, *WOX6*, *WOX11* and *YUCCA1* between 17-19 ℃ and 28-30 ℃. Values are mean ± sd (*n* = 3). The statistical significance is determined by Student's *t*-test; *, *P* < 0.05, **, *P* < 0.01, and ns represents no significance.


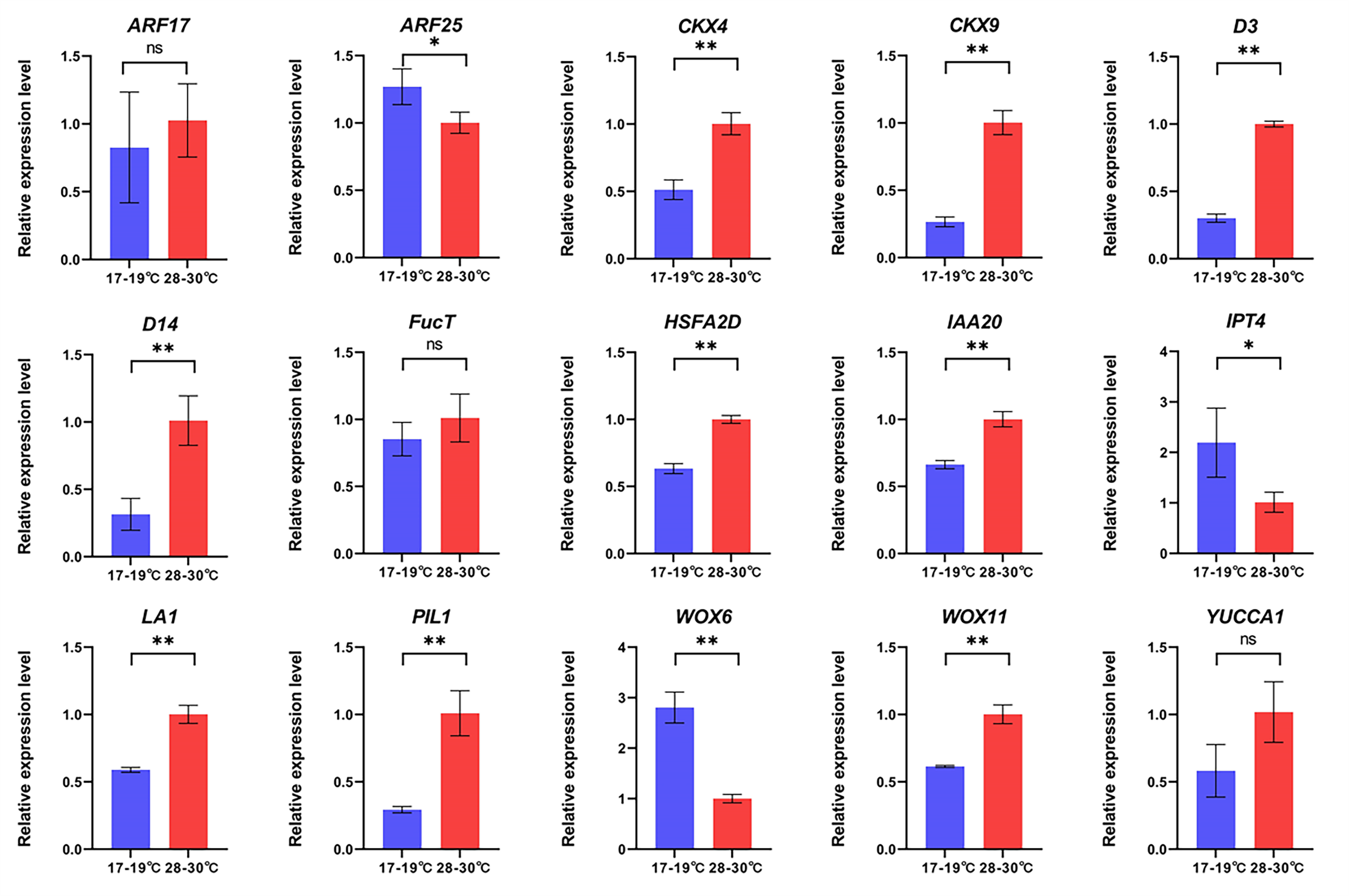


**Fig. S13** The expression levels of *IAA20*, *WOX6* and *WOX11* in upper and lower sides of rhizome. **A** The expression levels of *IAA20* in upper and lower sides of rhizome at 17-19 ℃ and 28-30 ℃. **B** The expression levels of *WOX6* in upper and lower sides of rhizome at 17-19 ℃ and 28-30 ℃. **C** The expression levels of *WOX11* in upper and lower sides of rhizome at 17-19 ℃ and 28-30 ℃*.* The 28-30 ℃ upper side of the rhizome (bud) is set as 1.00. Values are mean ± sd (*n* = 3). The statistical significance is determined by Student's *t* -test; ** *P* < 0.01.


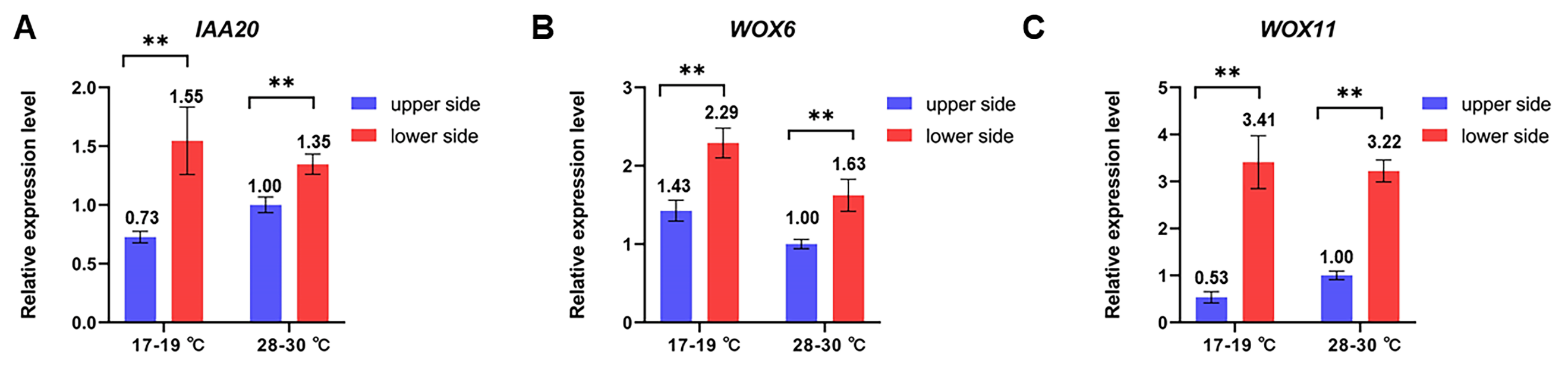


**Fig. S14** Expression of *IAA20*, *WOX6* and *WOX11* between upper and lower sides of rhizome (bud). **A** The start of upward growth of rhizome (bud) at 17-19 ℃. **B** The expression levels of *IAA20* in upper and lower sides of rhizome at 17-19 ℃ and 28-30 ℃. **C** The expression levels of *WOX6* in upper and lower sides of rhizome at 17-19 ℃ and 28-30 ℃. **D** The expression levels of *WOX11* in upper and lower sides of rhizome at 17-19 ℃ and 28-30 ℃*.* The upper side of the rhizome (bud) at 28-30 ℃ is set as 1.00. Values are mean ± sd (*n* = 3). **E** The ratio of *IAA20* expression level between the upper and lower sides of the rhizome (bud). **F** The ratio of *WOX6* expression level between the upper and lower sides of the rhizome (bud). **G** The ratio of *WOX11* expression level between the upper and lower sides of the rhizome (bud). Values are mean ± sd (*n* = 9). The statistical significance is determined by Student's *t*-test; ** *P* < 0.01, and ns represents no significance.


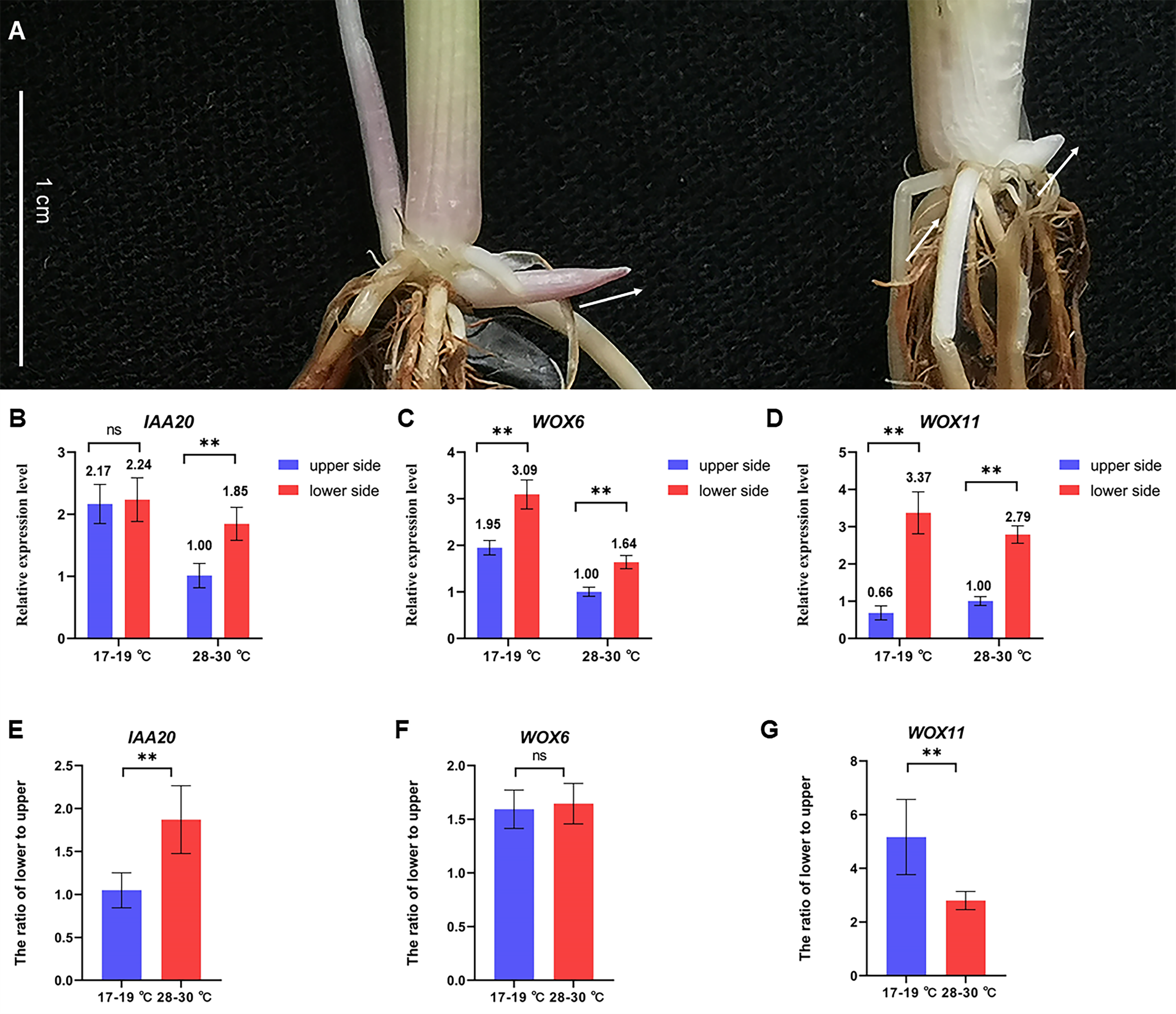

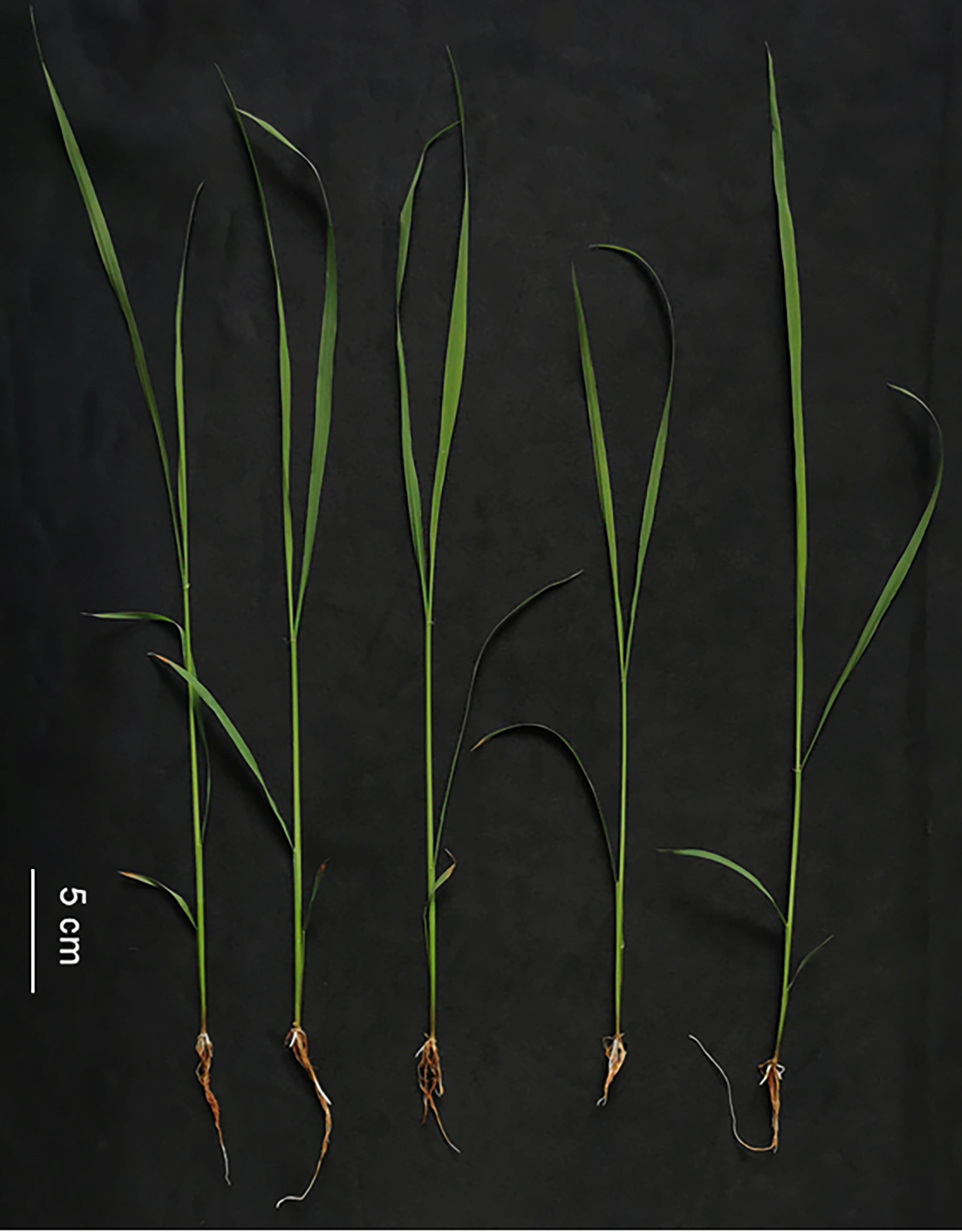


**Fig. S15** The *OL* seedlings prepared for different environmental temperature treatments.


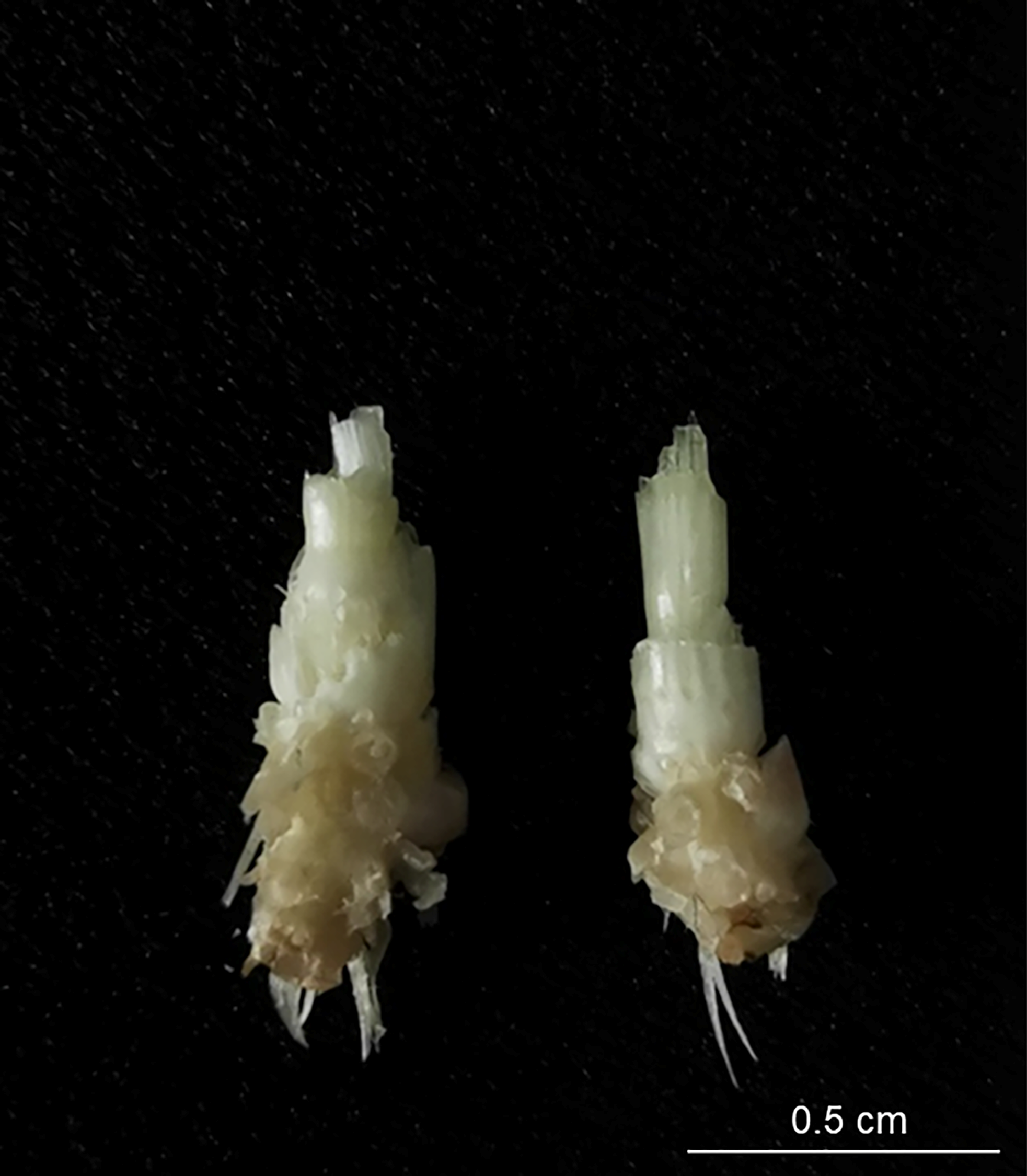


**Fig.** **S16** The crown of *OL* seedlings.
